# The Multifaceted Roles of Bovine Lactoferrin: Molecular Structure, Isolation Methods, Analytical Characteristics, and Biological Properties

**DOI:** 10.1021/acs.jafc.3c06887

**Published:** 2023-12-13

**Authors:** Tetiana Dyrda-Terniuk, Paweł Pomastowski

**Affiliations:** Centre for Modern Interdisciplinary Technologies, Nicolaus Copernicus University in Toruń, Wileńska 4, 87-100 Toruń, Poland

**Keywords:** bovine lactoferrin, bLF, iron-binding affinity, functional food formulations, biomedical applications, biological activity

## Abstract

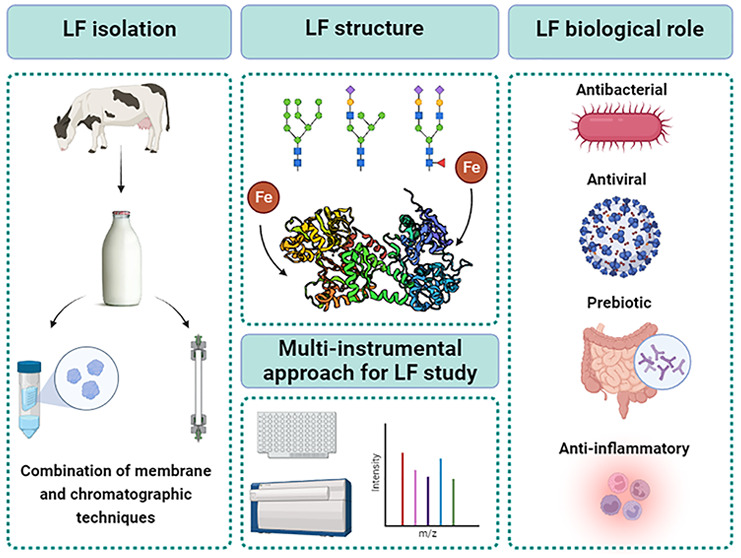

Bovine lactoferrin (bLF) is widely known as an iron-binding glycoprotein from the transferrin family. The bLF molecule exhibits a broad spectrum of biological activity, including iron delivery, antimicrobial, antiviral, immunomodulatory, antioxidant, antitumor, and prebiotic functions, thereby making it one of the most valuable representatives for biomedical applications. Remarkably, LF functionality might completely differ in dependence on the iron saturation state and glycosylation patterns. Recently, a violently growing demand for bLF production has been observed, mostly for infant formulas, dietary supplements, and functional food formulations. Unfortunately, one of the reasons that inhibit the development of the bLF market and widespread protein implementation is related to its negligible amount in both major sources—colostrum and mature milk. This study provides a comprehensive overview of the significance of bLF research by delineating the key structural characteristics of the protein and elucidating their impact on its physicochemical and biological properties. Progress in the development of optimal isolation techniques for bLF is critically assessed, alongside the challenges that arise during its production. Furthermore, this paper presents a curated list of the most relevant instrumental techniques for the characterization of bLF. Lastly, it discusses the prospective applications and future directions for bLF-based formulations, highlighting their potential in various fields.

## 1 Introduction

Whey proteins (WPs) are recognized as one of the most valuable components in milk whose characteristic feature is a wide spectrum of nutritional and health promoting effects. WPs are represented by a mixture of globular proteins, e.g. β-lactoglobulin (β-LG), α-lactoalbumin (α-LA), serum albumin (SA), immunoglobulins (Ig), lactoperoxidase (LPO), and lactoferrin (LF), dispersed in the continuous phase of the milk colloidal system which accounts for about 20% of the total protein fraction.^1^ On the other hand, whey poses a serious group of waste products with extremely critical levels of BOD (biological oxygen demand) and COD (chemical oxygen demand).^2,3^ Direct recovery and further supplementation of WPs could be a prospective approach for improving the functionality range of dairy products as well as for environmental protection.

LF is known as globular iron-binding glycoprotein which belongs to the transferrin protein family.^4^ Chelated ferric ions inside the LF structure ensure the characteristic salmon-pink color of a protein.^5^ Bovine lactoferrin (bLF) is predominantly found in the granules of neutrophils and mammary gland secretions, such as colostrum, transitional milk, and mature milk. The lactation stage is considered one of the most reasonable factors which strongly affects the composition of the cow’s milk. Significant changes of LF content are usually observed during the transition of colostrum (1.0–5.0 mg/mL) into mature milk (0.02–0.2 mg/mL).^6−8^ Remarkably, the amounts of lactoferrin and transferrin are relatively similar (LF, 0.83 mg/mL; TF, 1.07 mg/mL) in the colostral phase; however, their concentrations sharply decrease in the lactation period (LF, 0.09 mg/mL; TF, 0.02 mg/mL).^9^ Since LF is known as a biomarker of inflammation processes, accurate protein detection is extremely important for the monitoring of cattle’s health issues. For instance, the positive correlation between LF and immunoglobulin concentrations in serum is directly related to mastitis in cows.^10^

**Figure 1:**
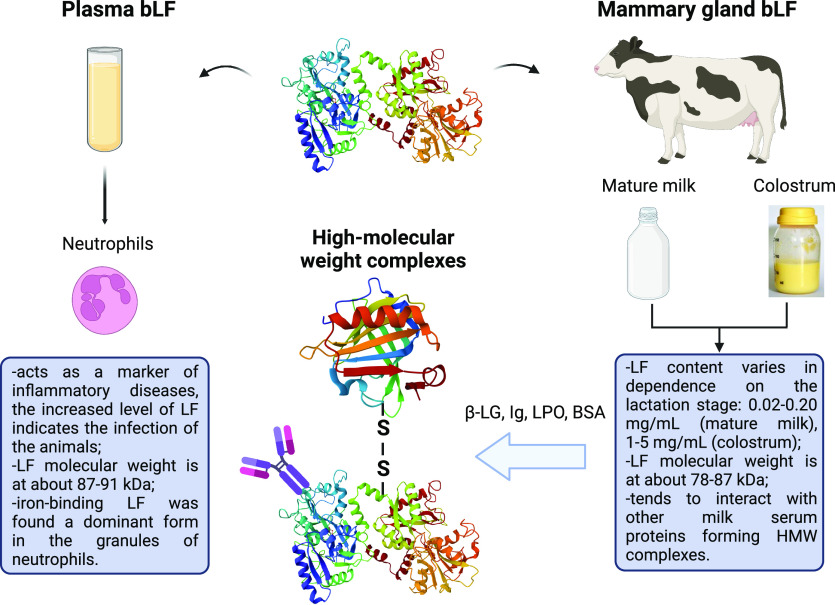
Comparison of bLF proteins from different sources of origin. Created with BioRender.com.

The molecular weight of LF might differ in dependence of its source of origin (Figure 1). It has been identified that LF mass from milk and colostrum was found in the range 83–87 kDa, while in neutrophils it was about 87–91 kDa. However, a single protein band appeared in both protein samples at 77 kDa resulting from the partial digestion of LF by *N*-glycanase.^11^ Thus, based on the LF source, it might be present in different molecular forms whose size is highly influenced by the nature (heterogeneity) of glycosylation. The major factors that might induce variations in LF glycosylation are the stage of lactation, age, breed of cow, season, and feeding. For instance, Barboza reported that the degree of glycosylation in human milk decreased after 2 weeks of lactation since colostrum was changed into mature milk.^12^ In addition, Jia et al. observed the same tendency in the case of LF in bovine milk; thus the amount of *N*-glycans content decreased in the following order of lactation stages: colostrum > transitional milk > mature milk.^13^ Despite the monomeric form, bLF, the protein also may occur as high molecular weight complexes (HMW-LF). This phenomenon was especially abundant in nonlactating mammary secretions (Holstein cows).^14^ It was indicated that LF trimers (*M*_w_ ∼ 250 Da) had much higher thermal stability as well as resistance to proteolysis than apo-LF and holo-LF.^15^ The structural stability of HMW-LF is probably caused by the structural integrity of larger oligomers. Besides, Ebrahim et al. showed that such HMW-LF complexes were sensitive to the addition of highly concentrated electrolyte solution (1 M NaCl) and dissociated into smaller structural units.^15^ LF is also capable of interacting with other proteins of milk, forming heterogenic protein complexes of LF–Ig, LF–CN, LF–SA, and LF−β-LG.^14,16^ Such bindings are characterized as noncovalent interactions which have a positive impact on the biological activity of the native LF molecule, enhancing some of its primary functions. Thus, Stephens et al. reported a higher antibacterial activity of LF–Ig complexes against *Escherichia coli* strains compared to LF alone.^17^

Until now, isolation of LF has been successfully performed from different mammals, including human, bovine, buffalo, goat, sheep, pig, horse, mouse, camel, and elephant. As it was reported, LF derived from human milk revealed the lowest similarity in sequence homology (>70%) in comparison to LF isolated from milk of other species.^18^ The difference in amino acid composition has a significant impact on the conformation stability of the biomolecule and other physicochemical properties (molecular mass, isoelectric point, thermal resistance).^19^ Currently, lactoferrin is a subject of great interest for researchers due to its high biological potential. LF is increasingly characterized as a multifunctional ingredient; it possesses antimicrobial, antiviral, immunomodulatory, and anticancer and prebiotic activities, which make it one of the most attractive candidates in the biomedicine and biotechnology areas.^20^ LF as an iron carrier avoids the formation of insoluble aggregates of ferric hydroxide; therefore, it might be safely applied in the treatment of anemia, especially in the case of pregnant woman.^21^ Previously, it was reported that 1.4 mg of iron might be maximum chelated per 1 g of protein, whereas the absorption of microelements by an organism is influenced by numerous factors, such as the daily diet, protein saturation state, pH conditions, human age, and physiological conditions.^22^ The additional advantage of LF’s iron-binding capacity relies on its antioxidant effect through the prevention of lipid peroxidation and the potential formation of reactive oxygen species (ROS) as a result of the Fenton reaction.^23^ The functionality of bLF can be developed as a result of interactions with other molecules (ligands), e.g. metals, vitamins, polyphenols, DNA, and lipopolysaccharides (LPS). Recently, interest in the synthesis of biologically active complexes based on bLF and metal ions has been growing. Such biologically active preparations allow for enhancement of beneficial and food values, especially in dairy products.^24^ For example, Hinrichs et al. pointed out that cheese enrichment in whey proteins is a novel approach in the development of functional food.^25^ Nevertheless, it is worth knowing that the lactoferrin incorporation will not only improve the nutritional quality and the functional properties of the final product; it will also require the reconstruction of the entire technological line.

Numerous health benefits and a wide spectrum of potential applications led to the significant growth of the demand for lactoferrin. The global production of bovine lactoferrin is expected to exceed a compound annual growth rate (CAGR) of 7.2% until 2028.^26^ Currently, to the list of key LF manufacturers joined the following companies: Glanbia Nutritionals (Kilkenny, Ireland), Synlait Milk (Dunsandel, New Zealand), Fonterra (Auckland, New Zealand), Merck (Darmstadt, Germany), Milei GmbH (Leutkirch, Germany), and Tatura Milk Industries Ltd. (Tatura, Australia) as a part of the Bega Group (Bega, Australia). The market statistics accounts the biggest application share of lactoferrin to infant formula, followed by dietary supplements and pharmaceuticals. One of current problems relies on the finding of an efficient and selective isolation method which will enable large-scale production of biologically active lactoferrin. The amount of LF found in bovine milk is relatively low (about 1%) in comparison to other WPs. Hence, LF isolation might be a quite challenging and time-consuming process. The first successful purification of bovine LF was performed in 1960 by Groves using DEAE cellulose chromatography.^27^ Comprehensive study of the nature of LF interactions has initiated the development of more advanced techniques. Traditional methods of ion-exchange chromatography, affinity chromatography, and membrane filtration have been improved by using more effective sorbents and modified membranes.^1,28^ In recent years, several complex techniques were investigated, including electrodialysis with a filtration membrane and magnetic affinity separation.^29^

This review presents recent advances in the structural characteristics of the LF molecule, such as glycosylation and metal-binding properties, describes the novel methods of protein isolation, and clarifies the essential role of biomolecules in disease treatment.

## 2 LF Structure

The three-dimensional structure of bLF is generally described as a single polypeptide chain containing 689 amino acid residues folded into two symmetric homogeneous lobes (N-lobe 1–341 and C-lobe Tyr342–Arg689) which are linked by three-turn α-helix (12 residues long from 333 to 344).^30^ According to crystallographic studies, each lobe is divided into two domains (N_1_/N_2_ and C_1_/C_2_). Although, the overall bLF structure has been known for a long time, it is worthwhile to mention LF genetic conservation/variation phenomena across different species. The LF gene is expressed in many species, e.g. human, bovine, buffalo, mouse, caprine, deer, equine, murine, and porcine. The protein genes from the transferrin family are typically composed of 17 exons (N-lobe, 2–8; C-lobe, 9–16, hinge region was at exon 9) whose lengths vary in the range 23–35 kb.^31^ Remarkably, it was found that 15 exons were identical between bovine and human lactoferrins, while the remaining two were responsible for gene differentiations that induced the variations in amino acid sequences.^32^ Seyfert et al. suggested that exons 4 and 12 play a significant role in the LF iron-binding properties and glycosylation.^31^ According to Wang et al.’s results, bovine and deer lactoferrins share 92% sequence homology.^33^ The authors particularly highlighted the difference in the amino acid compositions of two major antibacterial peptides—lactoferricin and lactoferrampin. Numerous molecular studies confirmed that genetic polymorphism is able to affect the protein conformational stability, the tendency to aggregate, and the biological activity.

The metal binding site is localized deeply inside the interdomain clefts of each lobe; thus one LF molecule is able to bind a maximum of two ferric ions. Interestingly, the binding cleft N-lobe reveals a more open conformation in comparison to the C-lobe.^34^ A slight difference in their flexibility is caused by the presence of a disulfide bridge formed between residues 483 and 677 which restricts additional movements in the C-lobe.^34^ Based on this, the metal binding capacity will differ between the N-lobe and the C-lobe. According to the iron saturation level, LF can be classified as apo-LF (saturation level <5%) and holo-LF (bound with two ferric ions). Native bLF is an intermediate state between apo-LF and holo-LF and accounts for approximately 15–20% of metal saturation.^35^ Native bLF is naturally composed of apo-LF, holo-LF, and monoferric LF forms which are present in whole milk in different proportions.^36^ The level of iron saturation has a great impact on the biological properties of a protein. For example, microbiological studies showed that apo-lactoferrin effectively inhibited the growth of pathogenic bacteria strains (*E. coli*, *Staphylococcus aureus*, *Pseudomonas aeruginosa*, *Mannheimia haemolytica* A2), whereas the supplementation of holo-lactoferrin did not reveal any antibacterial effect.^37^ In contrast, the supplementation of holo-LF ensured a more efficient cellular iron uptake, probably due to conformational specificity of the iron-saturated protein.^38^ Traditional methods of protein detection (ELISA, HPLC) and iron determination (UV–vis, ICP-MS) do not solve the problem of distinction and quantification of each of the present forms.^39^ Alternatively, Bokkhim et al. applied DSC studies to find the correlations between the iron saturation level and the thermodynamic parameters of protein denaturation.^40^ However, the suggested approach had a limitation as the iron content exceeded 75% because of the similarity in thermal behaviors of mono- and diferric bLF. The results of cation-exchange chromatographic separation performed by Makino et al. showed the prevalence of apo-LF and monoferric LF fractions in native hLF.^36^ Additionally, these forms might be distinguished based on their charge surface properties. For example, Kumar et al. observed three individual bands assigned to apo-LF, holo-LF, and monosubstituted LF which were present in buffalo LF samples. Results indicated a faster electrophoretic mobility as the level of iron saturation in protein increased.^41^

**Figure 2:**
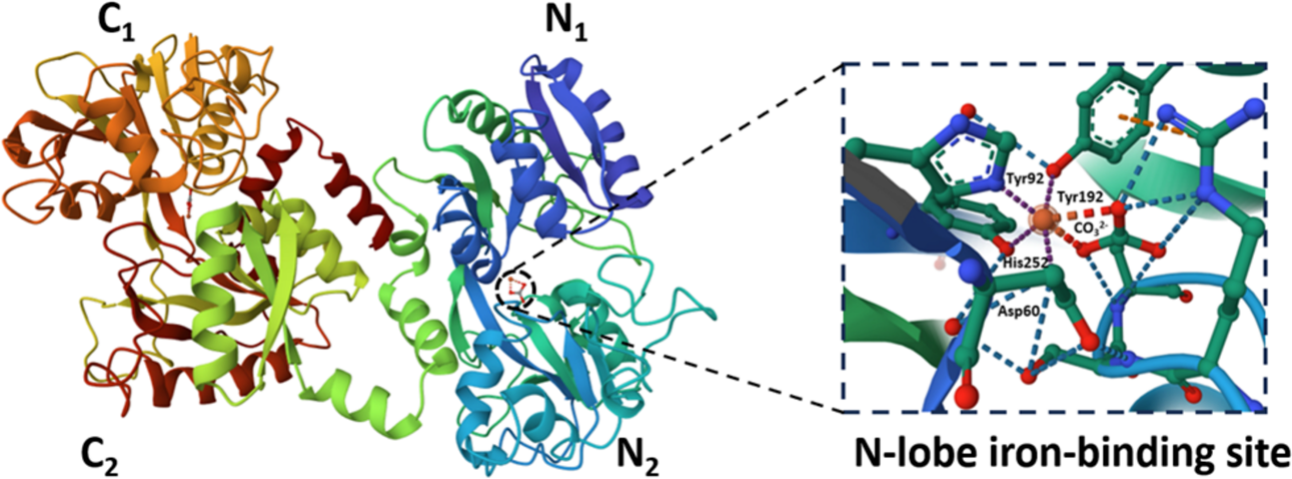
Three-dimensional structure of diferric bLF (Protein Data Bank code 1BLF).

Specific amino acid sequences and their particular arrangement promote an LF high binding affinity toward ferric ions (*K*_A_ = 10^20^).^42^ As a rule, iron complexation is accompanied by coordination of the bicarbonate ion, which plays a fundamental role in holo-LF stabilization. The iron-binding site has an octahedral geometry in which the ferric ion is chelated by the imidazole ring of histidine (His253 in N-lobe, His595 in C-lobe), phenolate oxygens of two tyrosinases (Tyr92 and Tyr192 in N-lobe and Tyr433 and Tyr526 in C-lobe), the carboxylate of aspartate (Asp60 in N-lobe, Asp395 in C-lobe), and two atoms of oxygen from the bicarbonate ion (Figure 2). Remarkably, bicarbonate incorporation is mediated by interactions with the arginine side chain and hydrogen bonding with threonine and the N-terminus of helix 5 in order to maintain a higher protein conformational stability.^43−45^ In the absence of metal, the conformation of the LF binding site is retained by intramolecular interactions, including the formation of hydrogen bonding between the pair of side tyrosine chains and van der Waals forces between histidine and aspartic acid. Typically, the metal saturation is initiated by insertion of a bicarbonate ion and followed by the attachment of two tyrosine residues. In the final step, iron coordination is completed by chelation with histidine and aspartic acid residues.^46^ The coordination bonding lengths range from 1.9 to 2.1 Å.^47^ Nevertheless, the results of LF tryptic digestion performed by Rastogi et al. showed that the binding site located in the C-lobe fragment was slightly deformed from an ideal octahedron at pH below 6.8.^48^ The geometry of the binding site is mainly influenced by the iron saturation level. The authors have demonstrated that gradually lowering the pH of the solution provides weakening of the bond strength between the ferric ion and the N^ε2^ imidazole atom of histidine due to enhancing the repulsion forces between these atoms; thus the coordination distance between these atoms tends to be increased. Currently, in the PDB database there are published crystallized structures of apo-LF isolated from human, equine, and camel species, while holo-LF was analyzed from human, bovine, buffalo, and equine organisms. Remarkably, the crystallization of native LF (mixture of apo- and holo-LFs) has not been performed yet. As it was reported, the metal content has a great impact not only on the geometry binding site but also on the protein conformation in general. The iron-free human LF revealed a more open conformation in contrast to the iron-saturated form. What is important is that the N-lobe undergoes loosening in apo-LF, while the C-lobe constantly remains rigid even after the metal release.^34^ The exact reason for that structural singularity of the C-lobe is still in question. Despite this, it would interesting to compare the behaviors of both lobes in the LF native state. The conformational changes induced by iron binding might vary among LF different species. For example, camel apo-LF possessed both open-structured lobes, thereby showing a higher conformational similarity with apo-ovatransferrin than with other lactoferrins.^49^ In contrast, the three-dimensional structure of mare apo-LF confirmed the presence of both N- and C-lobes in the closed state.^50^ The authors explained that the phenomena might be influenced by a difference in the relative orientation between the N- and C-lobes and specific interdomain interactions within the protein structure; nevertheless, this issue requires a more detailed review in the future.

Although the classical assumption affirms the chelation of only two ferric ions per LF molecule, several studies confirmed that the presence of additional binding sites on the protein surface. As it was shown, the protein modification at appropriate conditions, e.g. the molar ratio of reagents (bLF:FeCl_3_:NaHCO_3_), pH, and temperature, enables the formation of a supersaturated Fe–LF complex.^51^ According to results, a supramolecular structure of the synthesized complex was proposed in which LF molecules (15–16) were electrostatically linked through charged side chains of amino acids and Fe^3+^–HCO_3_^–^ ions. Alternatively, Nagasako et al. assumed that LF iron supersaturation might occur by electron donor–acceptor interactions with shares of cysteine, histidine, and tryptophan residues.^52^ Nonetheless, that question remains open for future research.

Iron exchange by other metals also may induce significant modification in the LF conformation. For example, replacement of ferric ions with copper in the N-lobe led to transformation of the binding site geometry to square pyramidal, resulting in the formation of hydrogen bonding between bicarbonate ion and tyrosine.^53^ Smith et al.^54^ studied lactoferrin complexes with different forms of vanadium (V^3+^, VO^2+^, VO_2_^+^). According to the results, metal chelation was mediated by the same amino acid residues as in holo-LF; however, the structural importance of bicarbonate ion decreased with the increase of the vanadium oxidation state.^54^ In contrast, the anion substitution of bicarbonate with the much bigger oxalate anion led only to slight changes of its arrangement in the binding site resulting in moving of the side chain of arginine away. Baker et al. also pointed out that the bigger size of substituted metal ions was characterized by lower affinity to LF.^55^ Thus, a comprehensive study of the bLF structure is crucial for characterization of the protein conformational stability, detection of the potential binding sites, and indication of the type of intra- or intermolecular interactions. Besides, it provides critical insights into the establishment of the relationship between the structure and functionality of biomolecules which is highly important for designing new drug candidates.

## 3 Factors Affecting LF Stability

It worthwhile to mention that LF conformation is characterized by high sensitivity to various physicochemical factors, such as pH, temperature, and ionic strength.^56^ Therefore, careful planning of the experiment by the selection of appropriate conditions is a necessary step for the protection of the biomolecule’s native structure and its primary biological function.

### 3.1 pH Conditions

LF stability and the level of iron saturation are tightly related to each other and are predominantly affected by the surrounding environment of the medium, especially pH. High iron-binding capacity is one of the fundamental features which contributes conformational stability of the LF structure over a wide range of pH. The nonchelated form of LF is considered more sensitive to unfolding at extremely low values of pH. In contrast, holo-LF was able to maintain ferric ions in the structure even at pH 2.^48^ LF desaturation is effectively carried out at pHs lower than 3.5, obtaining the protein with <2% metal.^57^ As expected, that pH lowering provides the opening of the LF conformation. The metal release occurred much slower from C-lobe than N-lobe binding site with pH lowering, which might be related to the higher rigidity of the C-lobe and its greater binding affinity to ferric ion.^43^ It has been demonstrated that iron dissociation begins at pH 6.7 in the N-lobe of bLF, while desaturation of the C-lobe was initiated at pH 5.6.^58^ A similar character of desaturation was observed in the case of lactoferrin isolated from human milk and neutrophils.^59^ Interestingly, in camel LF metal release in the C-lobe begins faster than in the N-lobe at pH 6.5 and 3.5, respectively.^49^ The stability of LF complexes might vary in dependence of the type of coordinated metal. For example, Jabeen et al. showed that Zn^2+^ dissociation from the C-lobe starts much lower (pH 4.6), in comparison to Fe^3+^ (pH 5.7).^60^ Importantly, that pH-induced LF desaturation is easily reversible and does not lead to the loss of primary protein metal-binding properties.^57^

### 3.2 Ionic Strength

Since LF dispersion represents a complex biocolloidal system, its stability is highly affected by ionic strength. The presence of co-ions induces some modification of the thickness of the electric double layer.^61^ Protein interactions with water molecules are strongly related to the type of present ions, which according to the Hofmeister effect might be classified as kosmotropic (cation, destabilizing; anion, stabilizing) and chaotropic (cation, stabilizing; anion, destabilizing). Sodium chloride is known, as a salt possessing a kosmotropic cation and a chaotropic anion, which tend to interact strongly with protein carboxylic and amino groups, respectively.^62^ Mela et al. reported that high electrolyte concentration (>30 mM NaCl) disrupts the balance between hydrophobic and electrostatic interactions in LF dispersion, leading to formation of negatively charged aggregates of apo-LF.^63^ These aggregates are described as micelles with bulk positively charged groups inside aggregates and more exposed to environment negatively charged groups. Conformational rearrangements were corresponding to significant changes of charge density on the protein surface, leading to reduction of the value of the LF isoelectric point from 8.4 to 6 at ionic strengths above 150 mM. Nilsson mentioned that LF conformation undergoes destabilization by aggregation and formation of amyloid fibrils at a relatively high NaCl concentration (150 mM) and heat treatment to 65 °C.^64^

### 3.3 Heat Treatment

Investigation of the heat stability of bLF is an essential step in technological processing which allows evaluation of the prospective of a macromolecule as a biologically active additive in new conceptions of food, cosmetic, and drug manufacturing processes. The treatment of extremely high temperatures provides significant conformational changes in the LF structure and subsequent loss of protein biological activity. It was shown that high temperatures are one the driving forces that facilitate the release of bound iron ions by weakening their interactions with LF binding sites.^65^ A previous study reported that LF thermal stability was positively correlated with the iron saturation level. Unsaturated bLF possess a greater heat sensitivity due to its less closed conformation, which makes its unfolding process more favored than that in holo-bLF. Bokkhim et al. has observed a tendency of apo-LF to denature at 70–71 °C, while the destabilization of holo-bLF occurred at 89–92 °C.^40^ Besides, the pH of the medium is an important factor that might contribute to LF thermostability. Remarkably, the apo-LF form was resistant to the formation of aggregates during ultra-high-temperature (UHT) processing at low ionic strength and acidic conditions. It was pointed out that pH 4 was the most optimal one allowing LF to maintain its conformation and biological activity under high-temperature processing.^66^ Heat treatment of bLF could induce a cleavage of disulfide bridges following by aggregation via thiol/disulfide interchange reactions. Many authors revealed that LF two-step aggregation corresponds to different thermal resistances of both LF lobes.^67^ Large insoluble aggregates of apo-bLF solution start to appear at 60 °C.^68^ Nevertheless, soluble disulfide-linked aggregates of holo-bLF (pH 6.6) were observed upon heating to 70–75 °C. These bands were more visible by application of SDS-PAGE in nonreducing conditions, because disulfide bridges were not cleaved.^16^ Difference in aggregate solubility is caused by the presence of ferric ion in the protein structure. Destabilization of iron-free LF occurs too fast compared to iron-saturated LF, favoring the formation of insoluble aggregates. Modification in the tertiary structure of bLF as well as an increase of particle size restricts protein interactions with bacteria membrane, thus its bacteriostatic capacity was reduced.

The highest iron-binding capacity was characterized at lower ionic strength below 0.01 M.^69^ Heating of LF above 80 °C at an ionic strength above 0.1 M decreased the level of iron saturation, precipitation, and partial unfolding. The authors reported that conformational changes involved breaking only noncovalent interactions (mainly hydrogen and electrostatic) resulting in releasing ferric ion. Heating over 72 °C provides only minor changes in the secondary structure of native LF (pH 7.5).^70^ Simulation MD studies showed inconsiderable changes in the contents of different types of secondary structure in LF. These minor modifications mainly concerned a partial reduction of the number of hydrogen bonds resulting in partial unfolding of α-helix. The β-sheet structure was less sensitive; thus the content of this structure remained constant during thermal processing. However, the percentages of random coils and turns in the LF structure were slightly increased.^42^ On the contrary, Iafisco et al. showed that thermal denaturation of LF was accompanied by reduction of α-helix and a significant increase of β-sheet content.^71^ Goulding et al. showed a similar effect of the irreversible transition of α-helix into β-sheet structure by using a high temperature–short time (HTST) thermal process.^72^ Thus, the composition of the secondary structure of bLF may vary with regard to the applied method of heat treatment. The temperature of denaturation and enthalpy change of denaturation of holo-LF is much higher than that of apo-LF, which suggests greater thermal resistance of iron-saturated forms of protein.^19,73^ Application of high hydrostatic pressure as and alternative to heat treatment also confirmed much evident conformation stability of iron-saturated LF at extreme conditions compared to metal unsaturated LF.^74^

Interestingly, relatively low temperatures are also capable of modifying protein biological functions. It was observed that LF storage of at 20 °C for 6 months significantly influenced the protein immunomodulatory function. The level of secreted tumor necrosis factor-α (TNF-α) decreased after hLF exposure.^75^ TNF-α plays a crucial role in the regulation of pro-inflammatory cytokine secretion and macrophage differentiation providing more effective protection of the organism against infections.^76^ Therefore, the consumption of fresh milk is extremely important in the case of infants since it allows preserving the beneficial properties of the product.

## 4 Isolation of bLF

LF isolation at a high level of purity might be a challenging task since its total amount in milk is negligible compared to other whey proteins.^77^ Currently, no standard procedure of lactoferrin isolation is established (Table 1). It worth noticing that the selected conditions of LF extraction might affect the yield, purity level, and biological activity of the final product. Main factors that might induce the modifications in each of four levels of the protein structure are included: extremely low pH, high temperature, pressure, and salt concentrations, and addition of chemical denaturants. The mentioned factors could lead to breakdown of intermolecular forces (hydrophobic, ionic, hydrogen, disulfide). For instance, Franco et al. reported that the thermal denaturation of lactoferrin occurs in the range from 60 to 90 °C in dependence on the saturation state.^78^ Remarkably, thermal processing at high temperatures is frequently accompanied by irreversible denaturation and as a result the complete loss of the functionality of the biomolecule.^79^ As it was previously reported, high pressure processing (>400 MPa) is capable of modifying the surface charge reducing the electrostatic repulsions between protein particles and facilitates the formation of aggregates.^80^ A preliminary purification procedure involves a few pretreatment steps, the number of which depends on the type of raw material (whole milk, colostrum, sweet cheese whey, acid whey). Traditionally, whole milk is subjected to centrifugation to receive a skimmed milk. The whey fraction is obtained by removal of casein micelles from skim milk resulting in acidic precipitation at pH 4.6.^81^ Ion-exchange chromatography is considered one of the most integrated techniques of LF isolation. The major principle of protein fractionation is based on a significant difference of isoelectric points between LF (pI 7.8–8.8) and the rest of the whey proteins, including β-LG (pI 4.9–5.4), α-LA (pI 4.4–5.1), and BSA (pI 4.7–5.2).^1,3^ The separation process is accompanied by strong interactions of LF with the stationary phase, while other proteins are easily eluted from the column.

**Table 1:** Isolation Approaches of LF from Different Sources

method of LF isolation	LF source	process	ref
cation-exchange chromatography by CM resin	bovine colostrum	First, milk must be defatted by centrifugation. Second, the casein fraction is removed from skimmed milk by acidic precipitation. Subsequently, the solution is neutralized by addition of NaOH. Some additional proteins were precipitated (salted out) by application of ammonium sulfate. Lactoferrin purification was carried out by the use of carboxymethyl Sephadex-C50 column and 0.2 M phosphate buffer (pH 7.7) as mobile phase.	(102)
combination of ultrafiltration with strong cation-exchange chromatography	bovine colostrum	A two-step ultrafiltration process was performed on a tangential flow filtration (TFF) model system with PLC membranes (nominal molecular weight cutoffs for each step were 100 and 10 kDa, respectively). The obtained retentate was adsorbed on a strong ion-exchange SP-Sepharose fast flow column and eluted with buffer solution composed of 0.05 M sodium phosphate with 0.1–1.0 M NaCl (pH 7.7).	(100)
ion-exchange chromatography on monolithic columns	acid whey	Preliminary microfiltration of whey was performed with ceramic alumina/zirconia asymmetric membranes (pore diameter < 0.8 μm). The obtained permeate was loaded on a CIM monolithic ion-exchange column. Selective LF isolation was related to its higher pI value in comparison to other whey proteins. Absorbed LF was eluted with a relevant buffer solution. A TFF system was applied in order to desalt and concentrate the obtained LF fraction. Lastly, the protein was freeze-dried.	(73)
cross-flow microfiltration coupled with isolation on strong cation-exchanger membranes	sweet cheese whey	Whey cheese was subjected to cross-flow microfiltration with use of a Microdyn tubular system (spun with polypropylene filter cartridge) in order to eliminate insoluble particles (lipids, casein). Subsequently, LF isolation from permeate was performed by application of the strong cationic membrane adsorber Sartobind S. The elution process was carried out in a three-step operating sodium chloride salt gradient. Finally, the obtained eluates were desalted using Sartocon II with cellulose acetate membrane (area of 0.7 m^2^ and a cutoff of 30 kDa) and lyophilized.	(103)
electrodialysis with filtration membrane	whey protein isolate	LF undergoes aggregation in a salty SMUF buffer, such as that available naturally in whey. The solution containing the micelles of lactoferrin and other dairy proteins can then be separated by electrodialysis with a filtration membrane (PVA glutaraldehyde as a cross-linking agent, catalyzed by sulfuric acid), where proteins with smaller size will pass through, while the larger aggregates of will be retained.	(104)
separation on magnetic affinity adsorbents	acid whey	Preliminary, skimmed milk obtained by centrifugation was acidified in order to eliminate casein. Remained whey proteins were incubated together with PGMA–NH_2_–heparin magnetic particles at 10 °C in an ice bath for 2 h. Subsequently, particles were removed from the reaction mixture by use of PBS buffer solution (pH 6) as eluent. The release of LF from magnetic affinity adsorbents was conducted by elution with 0.5 M NaCl.	(29)
affinity chromatography equipped with immobilized Cibacron Blue F3GA dye column	bovine colostrum	Colostral milk was subjected to the traditional procedure of sample pretreatment, including defatting and casein removal. Subsequently, whey was slightly alkalized to pH 6 by the addition of sodium hydroxide. The LF fraction was separated on a Cibacron Blue F3GA column using 0.1 M NaOH as eluent. The efficiency of the proposed methodology was related to strong electrostatic interactions between the anionic stationary phase and positively charged LF molecules.	(105)

In neutral pH positively charged LF has a much higher affinity to a cation-exchange resin. Anionic sorbents containing strong sulfopropyl (SP) and weak carboxymethyl (CM) functional groups belong to the most commonly used resins in cation-exchange chromatography.^82^ The adsorption behavior and the strength of the interaction of the target protein with the stationary phase could be modified, controlling the surface charge of the biomolecule. Remarkably, Voswinkel et al. paid attention to that the binding affinity of LF to adsorbent is highly dependent on the protein saturation state.^83^ According to results, apo-LF was more sensitive to applied separation conditions and was characterized by a lower adsorption capacity compared to holo-LF, especially at the low pH range. It might be related to the structural singularities of both LF forms and the difference in distribution of the surface charge. As previously mentioned, apo-LF is characterized by a more “open” conformation; therefore, it is possible to suggest that it has more accessible ionizable groups in contrast to holo-LF. The presence of minor amounts of lactoperoxidase (LPO) molecules (pI 9.3–9.6) and immunoglobulins (5.5–8.3) might become a serious hardship in the production of highly purified biologically active LF by ion-exchange chromatography.^84^ LPO is known as a minor whey enzyme mainly involved in the immune defense function and whose content in bovine milk varies from 15 to 50 mg/L.^3^ The problem of LPO elimination could be solved by selection of the appropriate isolation conditions, e.g. flow rate of the mobile phase, buffer pH, and ionic strength.^85^ Uchida pointed out that the efficiency of separation of LF from LPO might be improved by use of a buffer solution with pH >5 and ionic strength higher than 0.5.^86^ For instance, Ng et al. applied two-step isocratic elution with 0.4 and 0.6 M sodium chloride buffer (pH 7) for fractionation of an LF–LPO mixture.^87^ The detection of LPO is frequently performed with UV–vis analysis by measuring the maximum absorbance in the Soret region at λ_ex_ 412 nm.^82^ Negligible amounts of lysozyme (0.15–2.7 μg/mL) also might constitute an interfering factor (pI 11) during LF isolation.^88,89^ In this case, the protein separation was performed by use of UF zirconia membranes, modified with charged cationic (ethylenediamine, EDA) groups.^89^ Interestingly, Lech et al. has detected the presence of serum albumin after goat whey treatment on a CM-Sepharose column.^90^ Thus, effective separation of both proteins was achieved by anion-exchange chromatography with a DEAE-Sepharose resin.

Affinity chromatography is another separation technique frequently used for the purification of whey proteins. The stationary phase is composed of immobilized antibodies (ligands) that selectively interact with the target molecule, while other components are easily eluted from the column. Sepharose modified with heparin, metal ions, oligonucleotides, monoclonal antibodies, organic dyes, and even other whey proteins was mentioned as an effective adsorbent for LF molecules.^12^ Lampreave et al. showed that LF is favored to form noncovalent associations with whey proteins, in particular with β-LG.^91^ Therefore, the immobilization of insoluble β-LG particles might be a potential approach for the successful isolation of LF.^92^ Unfortunately, this method is insufficient for bovine milk matrix since it contains great amounts of β-LG which will also compete with LF for binding sites.

In recent years, membrane technology has been becoming more attractive and rapidly developed in food production, especially in the dairy sector. Membranes with specific pore sizes are widely used during diverse technological processes, e.g. microbial decontamination, defatting (microfiltration, MF), concentration and fractionation of whey proteins (ultrafiltration, UF), removal of low-molecular compounds, mainly salts, lactose, and peptides (nanofiltration, NF; reverse osmosis, RO).^93^ LF purification by the membrane separation approach is not implemented to such an extent as chromatographic technologies, but it also has a great perspective of application in large-scale production. To the major advantages of membrane processing should be included less impact of adsorption and diffusion effects during mass transfer in comparison to chromatographic methods.^94^ In addition, it enables high separation effectiveness and minimizes the risk of destruction, since samples are subjected to low-temperature conditions.^95^ Unfortunately, membrane separation becomes challenging in the case of macromolecules with similar molecular weights (such as BSA, 65–69 kDa; LPO, 77–90 kDa; and LF, 78–84 kDa).^96^ The selectivity of membrane adsorption technologies might be improved as a result of chemical modification of the membrane surface and attachment of specific functional groups. One such approach introduced the separation of milk proteins according to their electric properties with the use of charged membranes.^97^ Basic principles assume that proteins with the same net charge as the filtration membrane are retained due to electrostatic repulsions, while components having neutral or opposite-charged surfaces easily pass through the membrane barrier.^98^

Valiño et al.^97^ showed that pH manipulation allowed for the effective separation of individual proteins in BSA and LF mixtures. In the experiment, the diafiltration was performed by using charge-modified membranes made of regenerated cellulose (100 kDa cutoff). The authors reported that the most optimal fractionation of BSA was performed at pH 5 with the use of a positively charged membrane; however, LF was successfully isolated by application of a negatively charged membrane at pH 9.^97^ On the other hand, the separation at such conditions might be inefficient because it is known that the pI of LF varies in a wide range from 4 to 10 in dependence of the source of origin, level of glycosylation, the iron saturation state, or instrumental approach.^99^ Internalization of ion-exchange groups to the membrane surface, similar to ion-exchange columns, provides improved separation of LF from other milk proteins. Such an approach allows operation at high flow rates and minimal diffusion effects. For example, Plate et al. have demonstrated the separation of the LF–LPO mixture from sweet cheese whey by a membrane adsorber modified with sulfonic groups (Sartobind S).^1^ It was revealed that LPO was gradually eluted by increasing the ionic strength from 0.1 to 0.2 M NaCl, while the LF fractionation required 1 M NaCl eluent. The obtained product was characterized by 95% purity. In contrast, Teepakorn et al. performed the purification of LF from BSA by use of monolithic columns equipped with cation-exchange membranes, the same as previously described.^95^ In order to improve the LF recovery, the number of membranes equipped into the column was increased to 33. Elution with phosphate buffered saline (pH 6) provided a sufficient adsorption of positively charged LF on the stationary phase with the complete removal of negatively charged BSA molecules. On the other hand, the application of columns with monolithic material might be problematic due to poor reproducibility as a result of pore size heterogeneity. The combination of membrane and chromatographic techniques represents another progressive trend in the isolation of milk proteins. Lu et al. proposed an efficient two-step methodology aimed at large-scale LF isolation from bovine colostrum.^100^ Primarily, colostral whey was subjected to two-step UF on regenerated cellulose membranes with cutoffs of 100 and 10 kDa, respectively. In the second step, LF was eluted from the obtained filtrate using a cation-exchange SP-Sepharose column and phosphate buffer (pH 7.7). Remarkably, the combination of microfiltration with affinity chromatography enabled obtaining LF product with a final purity of 95%.^101^ A stable complex formed between LF and heparin Sepharose resin was subjected to microfiltration to remove the unbound protein fraction and other impurities. Finally, the target protein release was conducted by changing the buffer ionic strength. Thus, it is worthwhile to highlight that one of the most substantial advantages of combined isolation techniques relies on the possibility to obtain the product with higher recovery and at a higher purity grade.

The extracted LF fraction is typically subjected to dialysis (cutoff 12–14 kDa) for several days to remove salts and other contaminants. Alternatively, it might be concentrated by using the cross-flow filtration system Sartocon.^1^ Drying is a key step in the final preparation of lactoferrin which relies on receiving the powdered product ready for packaging. Among the most popular drying methods widely utilized in lactoferrin preparation, freeze drying (lyophilization) and spray drying might be distinguished. Although previously it was mentioned that drying plays a negligible role in the structural stability and functionality of lactoferrin, the recent studies showed by Morel et al. proved the opposite effect.^106^ The authors noticed that the spray-drying approach was more destructive in the case of bLF samples inducing the denaturation of 14–17% of the structure, while freeze drying caused only 7% of damage. Such a difference might be related to the extremely high temperature applied during spray drying (>140 °C) in contrast to freeze drying (≤40 °C). What is important is that the selected method had no visible impact on the protein bacteriostatic activity. According to results obtained by Wang et al., the freeze-drying technique was much more effective in water removal; thus the determined moisture content was only 2.7% (w/w) while it was about 5–9% (w/w) in the protein after spray drying.^107^ Remarkably, the degradation effects in the lactoferrin structure were not much evident as in the previously mentioned study, and they varied in the range 0.9–2.0%.

## 5 Recovery of Waste Products

As previously mentioned, the total amount of LF in cow milk is trace and varies at about 0.01–0.05% (w/w).^108^ Waste products derived as a result of LF production constitute the additional milestone in the industrial sector. The main goal of this issue involves the maximum engagement of byproducts obtained at every single stage in order to reduce the effects of environmental pollution and prevent economic losses. Generally, dairy waste might be divided into two categories: effluent and sludge.^109^ Milk fat, separated during milk microfiltration, might be utilized as a crucial element in the formulation of various dairy products, e.g. cream, cheese, cream cheese, butter, and anhydrous milk fat (AMF).^110^ Interestingly, the milk fat content plays a significant role in the creation of unique organoleptic features (taste, texture, color) in the final product.^111^ The recovered casein is characterized by a high nutritional value, which enhances its attractiveness in food manufacturing. Casein produced by acid precipitation is insoluble in water; thus it is preferably dissolved by the addition of a strong alkali, forming caseinates.^112^ Due to its unique structure, casein is widely utilized for the delivery of numerous bioactive compounds, stimulating the development of novel dietary supplements or pharmaceuticals.^113^ The whey fraction remaining after LF isolation represents a rich source of high-value proteins, e.g. α-LA, β-LG, SA, and Ig. Whey proteins are characterized by rich contents of essential amino acids which are easily absorbed by organisms and stimulate muscle protein synthesis.^114^ Recently, the production of functional beverages fortified with whey proteins for athletes is gaining in popularity.^115^ The major challenge in the spreading of these beverages is focused on the selection of the appropriate packaging and storage conditions to ensure the maximum stability of whey proteins and to prevent their denaturation and the loss of biological activity.

## 6 The Role of LF–Metal Interactions

Shortly after the isolation, the native protein is subjected to some chemical modifications in order to obtain an LF form with a desired level of iron saturation. LF desaturation is traditionally performed by dialysis against citrate buffer at pH <3.5 and then against deionized water. The described process is known as reversible; thus the protein resaturation might be carried out by mixing of apo-LF with ferric nitriloacetate (Fe-NTA) and then against deionized water in order to remove unbounded ions.^35^ As it was shown in previous studies, LF is characterized by the greatest thermal and conformational stabilities among the rest of the proteins from the transferrin family.^116^ Besides, the native structure of bLF exhibits a relatively high resistance in the presence of proteolytic enzymes.^117^ Many authors directly associated that phenomenon with the conformational specificity of binding sites between LF and TF. The release of iron ions occurs 100 times slower in LF compared to TF. Binding clefts of apo-TF, as a rule, possess a more wide-open conformation being exposed to the aqueous environment. On the contrary, the binding site located in the C-lobe of LF favors maintaining the closed conformation even during desaturation; therefore, the protein contact with the polar medium is always partly restricted.^34^ LF saturation accompanies the closure of binding clefts which occurs in both lobes.^118^ The greater percentage of bounded metal provides the growth of surface tension of LF; thus the holo form of the protein achieves a more compact structure.^119^ In contrast, Bluard-Deconinck et al. did not notice the obvious difference in iron-binding capacity between released N- and C-fragments of LF.^120^ Indeed, Lin et al. have related the changes in metal complexation between C- and N-sites of apo-hTF with different rates of bicarbonate insertion to each lobe.^121^ Ferric ions exhibited a more visible binding preference to the C-lobe compared with the N-lobe as the concentration of bicarbonate in the solution decreased. Baker as also pointed out the importance of hydrogen interactions in the dilysine pair (Lys206–Lys296) in the TF structure and its susceptibility to iron release.^122^ Remarkably, based on previous studies, the presence of a more compact conformation of the N-lobe in rTF and hTF than in bLF was reported.^47^ In this case, the conformational variations between LFs from other species were related to the specificity of amino acid composition and lobe orientation, but not to iron-binding affinity.^47^ In addition to this, SDS-PAGE analysis confirmed a higher susceptibility of the bLF structure to trypsin digestion than bTF, showing a greater degradation effect after enzyme treatment.^123^ Comparing both iron-saturated proteins, 41% of TF remained undigested after 24 h, while in the case of LF it was only 6%. Regarding this, TF could be a more compatible candidate for cellular iron uptake in contrast to LF.

The metal incorporation mostly affects the LF tertiary structure; however, a slight alteration in the secondary structure is also present.^124^ Based on CD studies, the determined ratios of α/β structures at pH 7 were equal to 20.0/59.0 and 11.5/70.0 for apo-LF and holo-LF, respectively.^56^ Indeed, Xia et al. reported that the percentage contents of the individual secondary structures in apo-LF and holo-LF were relatively similar and ranged about 18–20% of α-helix and 53–55% of β-structure.^125^

A characteristic feature of LF among other whey proteins is a comparatively high isoelectric point. Based on the UniProt database, the percentage content of basic amino acids (14.66%) in the bLF polypeptide chain is higher than that of acidic amino acids (11.03%). The protein surface contains regions with high concentrations of positive charge, such as the N-terminal region where the majority of basic amino acids are located.^126^ Although the protein isoelectric point is directly related to the amino acid composition, the level of iron saturation also has a great impact on the physicochemical nature of the protein surface and the charge distribution.^127^ As a result, the isoelectric points of apo-LF and holo-LF can vary due to differing availability of charged amino acids.^30^ For example, Lys637 located in the C_1_ domain and the Arg463 and Lys544 residues of the C_2_ domain are deeply buried in holo-LF, although they became more exposed in the cleft of the apo form.^46^ Isoelectric points of native LF and holo-LF determined by potentiometric titration were found in the range 8.0–9.0 and dropped to 5.7, in the case of apo-LF.^119^ The major reason for such a broad pI range for LF proteinmight also be related to the type of applied instrumental method, e.g. isoelectric focusing (IEF), two-dimensional gel electrophoresis (2D-PAGE), capillary isoelectric focusing (cIEF), and potentiometric titration. For example, IEF performed by Voswinkel et al. showed a negligible difference between the pI values of apo-bLF and holo-bLF, which were in the ranges 9.3–9.4 and 9.4–9.5, respectively.^83^ However, the study of Yoshida et al. carried out with the same technique reported the cationic character of bLF, whose pI ranged from 4.8 to 5.3.^128^ Potentiometric titration is another popular technique widely used for characterization of electrochemical properties of biomolecules which is based on the measurements of zeta potential in the wide pH range by gradual addition of strong acid/basis. Many aspects might be important during the analysis, predominantly the type of dispersion medium, ionic strength and the protein concentration.^129^ For, example Pryshchepa et al. determined that the pI of bLF dispersed in 0.09% (w/w) sodium chloride solution at the concentration of 1 mg/mL was at about 7.4.^130^ However, Valiño reported that the pI of bLF suspension (1 mg/mL) in 0.01 M NaCl medium (which is equal to 0.06% (w/w)) rose to 9.2.^129^

Despite iron, several studies have confirmed the ability of LF to interact with other metal ions, including Cu(II), Zn(II), Mn(III), Co(III), Ti(IV), and Ag(I).^131,132^ It has been reported that iron substitution with other metals induced exclusively minor changes in the hLF structure; thus their binding mechanisms are considered to be similar. Nevertheless, among all of them, iron was characterized with the highest binding affinity, indicating that the formation of LF–Fe complex is the most favorable.^132^ Indeed, even inconsiderable conformational modifications of LF in protein structure might cause fundamental changes of its biological activity. The previously mentioned metals belong to d-block elements (transition metals) which are frequently described as essential mediators of various biological processes in the human organism. Iron, copper, manganese, zinc, and copper act as protein inorganic cofactors required for activation of enzymatic reactions and for maintaining the structural stabilities of biomolecules.^133^ Besides, many metal–protein complexes mediate in the regulation of cellular metabolism, oxygen transport, and prevention of DNA damage.^134^ Importantly, metals are also capable of promoting the process of protein glycosylation.^135^ For example, Prabhu et al.^135^ pointed out that manganese and iron ions might be involved in the regulation of glycosylation pathways since they induce the activation of glycosylation enzymes.^134^ However, the direct impact of these metals on the glycosylation profile of LF has not been discovered until now.

## 7 LF Glycosylation

Protein glycosylation is a post-translation modification which has a fundamental influence on the biological activity of a macromolecule in different aspects, including structural stabilization, folding process, proteolytic resistance, immune response, signal recognition, bacterial adhesion, and antiviral activity.^81^ Glycans are considered the markers of evolution, and their structures and diversity at each position are specific to each species and are influenced by different factors. Milk contains such glycosylated components as LF, κ-CN, immunoglobulins (IgG, IgA, IgM), α-LA, and LPO.^136,137^ Glycosylation plays a crucial role in the physicochemical characteristics of bLF, particularly the molecular weight and ionic charge. The molecular mass of bLF is variable throughout seasonality, the lactation period, and the cow’s feeding. All of the mentioned factors contribute to the difference in carbohydrate composition and glycosylation level of a biomolecule. For instance, the overall number of *N*-glycans has decreased from 41 to 22 after the transition of the colostrum into mature milk.^13^ Besides, the structural diversity of the detected glycans might be affected by the type of applied mass spectrometry technique (MALDI-TOF/MS, ESI-MS).

Glycans in proteins are composed of *N*-acetylglucosamine (GlcNAc), *N*-acetylgalactosamine (GalNAc), sialic acid (NeuAc), mannose (Man), galactose (Gal), and fucose (Fuc) monosaccharide residues. Monosaccharide units linked in a particular order represent a glycan chain. Generally, the N-type of glycosylation is only present in the case of the LF molecule, which implies that glycan chains are covalently attached to the nitrogen atom of the asparagine side chain via GlcNAc.^136^ The glycan core consists of two GlcNAc and three Man residues. Three types of N-glycosylation are distinguished, e.g. complex, hybrid, and high mannose. This classification is based on the carbohydrate moieties associated with the glycan core. The heterogeneity of glycan chains is predominantly affected by enzyme expressions in the bovine mammary gland (mainly, glycosyltransferases and glycosidases), lactation period, and availability of protein substrate.^12,138,139^ Complex and high mannose are the dominating types of glycosylation in the cow’s mature milk.^140^

Glycan content in the C-lobe was higher than that in the N-lobe, including three glycan chains.^47^ Asn368 is linked with one unit of GlcNAc and Asn476 is bounded to one unit of α-1,4-Man and two units of GlcNAc; however, the most glycosylated residue was Asn545, which is connected to four units of α-1,4-mannose and two units of of GlcNAc.^58^ Based on the number of glycosylation sites, LF can be classified into LF-a and LF-b. Four completely glycosylated distinct N-linked glycosites (Asn233 is located in the N-terminal while Asn368, Asn476, and Asn545 are found in the C-terminal) are present in LF-b.^141^ Despite all mentioned, LF-a has a fifth potential glycosite which is found at the Asn281 position. The LF-a form is more abundant in bovine colostrum than in mature milk (glycosylation levels are 30 and 15%, respectively).^142^ Both molecular forms might be separated by use of cation-exchange chromatography with carboxymethyl resin. SDS-PAGE electrophoresis verified a greater size of LF-a (84 kDa) in comparison to LF-b (80 kDa) as a result of additional glycan units at the Asn281 residue.^143^ The structure of the glycan chain linked to Asn281 was found to be heterogeneous involving GlcNAc, GalNAc, Man, Gal, and Fuc moieties.^142^ bLF-b was about 10 times less resistant toward trypsin digestion than bLF-a. Thus, the glycan chain at the Asn281 position may actively participate in the stabilization of the protein structure by limiting the availability of basic amino acid residues to enzymatic action.

Despite *N*-glycan attached at the Asn281 position, other chains also may contribute to the stability of the protein’s three-dimensional structure. Indeed, the glycan chain at Asn545 is arranged between two domains; thus it can easily participate in interactions with protein amino acids.^60^ It has been observed that this type of “protein–glycan” interactions prevents the release of metal ion at lower values of pH and protect bLF against proteolysis, enhancing the rigidity of the C-lobe conformation. On the other hand, Van Berkel et al. showed no difference in iron-binding capacity between glycosylated and nonglycosylated forms of hLF.^144^

The origin of LF is a crucial factor which affects the composition and heterogeneity of glycan chains. It was reported that bLF contained a high amount of sialylated *N*-glycans, while fucose residues occurred predominantly in hLF.^145^ The content of sialylated *N*-glycans was higher in bovine milk compared to human and goat milk.^93^ Sialic acid in the glycan chain might be responsible for the maintenance of metal in the protein structure. Desialation of human LF by neuraminidase treatment induced the alteration in the iron-binding capacity of bLF; thus the LF saturation level was 3-fold reduced.^146^ Interestingly, that resialation provided partial restoring of the initial amount of bounded metal. Besides, sialic acid is known as the only charged saccharide present in LF, which is traditionally linked in the terminal position of complex or hybrid *N*-glycans. Consequently, it can be assumed that these carbohydrate residues are capable of modifying the surface charge in glycosylated proteins. For example, Shimazaki et al. related the difference in pI values of bLF isolated from mature milk (pI 8.94) and colostrum (pI 8.3–8.52 and pI 8.18–8.32) with different contents of sialic acid in the protein.^99^ The phenomenon of charge heterogeneity observed in the pI of colostral LF also could be associated with some variations in the glycosylation profile. Importantly, the distribution of negative charge in bLF molecules was increased after the sugar release (deglycosylation). Analogically, Barrabés et al. noticed that a higher content of sialic acid has a tendency to shift the protein isoelectric point to lower values.^147^

The role of glycosylation in the protein structure is not completely understood until now. The monosaccharide diversity of LFs from different species may contribute to protein biological activity. In particular, this feature can influence the antibacterial potential of bLF. For example, Karav reported that the complete deglycosylation of LF significantly reduced its antimicrobial action against *E. coli* DH5a strains.^148^ It was shown that apo-LF-a had higher antibacterial activity compared to apo-LF-b, indicating the importance of additional glycan at Asn281.^143^ Various studies have noticed that sialic acid is capable of indirectly interacting with the outer membrane of Gram-negative bacteria. The highly acidic character of sialic acid ensures an effective chelating of calcium ions from lipopolysaccharides leading to destabilization of the bacterial membrane.^149^ On the other hand, it was shown that in the presence of diasialated hLF the adhesion of *Salmonella enterica* significantly increased.^12^ Deasialated bLF exhibits higher antiviral activity against rotavirus infection than native bLF. Treatment by deglycosylated and desialated bLF showed a more effective inhibition of influenza virus infection, especially at the initial step of viral invasion.^150^ It was suggested that deasilation facilitates the interaction between anionic cellular membrane and bLF; thus the protein becomes more competitive for host cells binding. Furthermore, sialic acid removal enhances the bLF–virus interactions which is leading to prevention of the binding of viral particle to cellular receptor.^151^ It was reported that the highly fucosylated *N*-glycan core in human milk positively influences the gut microbiota of infants, reduces the number of pathogen infections, and promotes the growth of beneficial *Bifidobacterium* and *Lactobacillus* strains.^152^

## 8 Multi-instrumental Approach for LF Characterization

**Figure 3:**
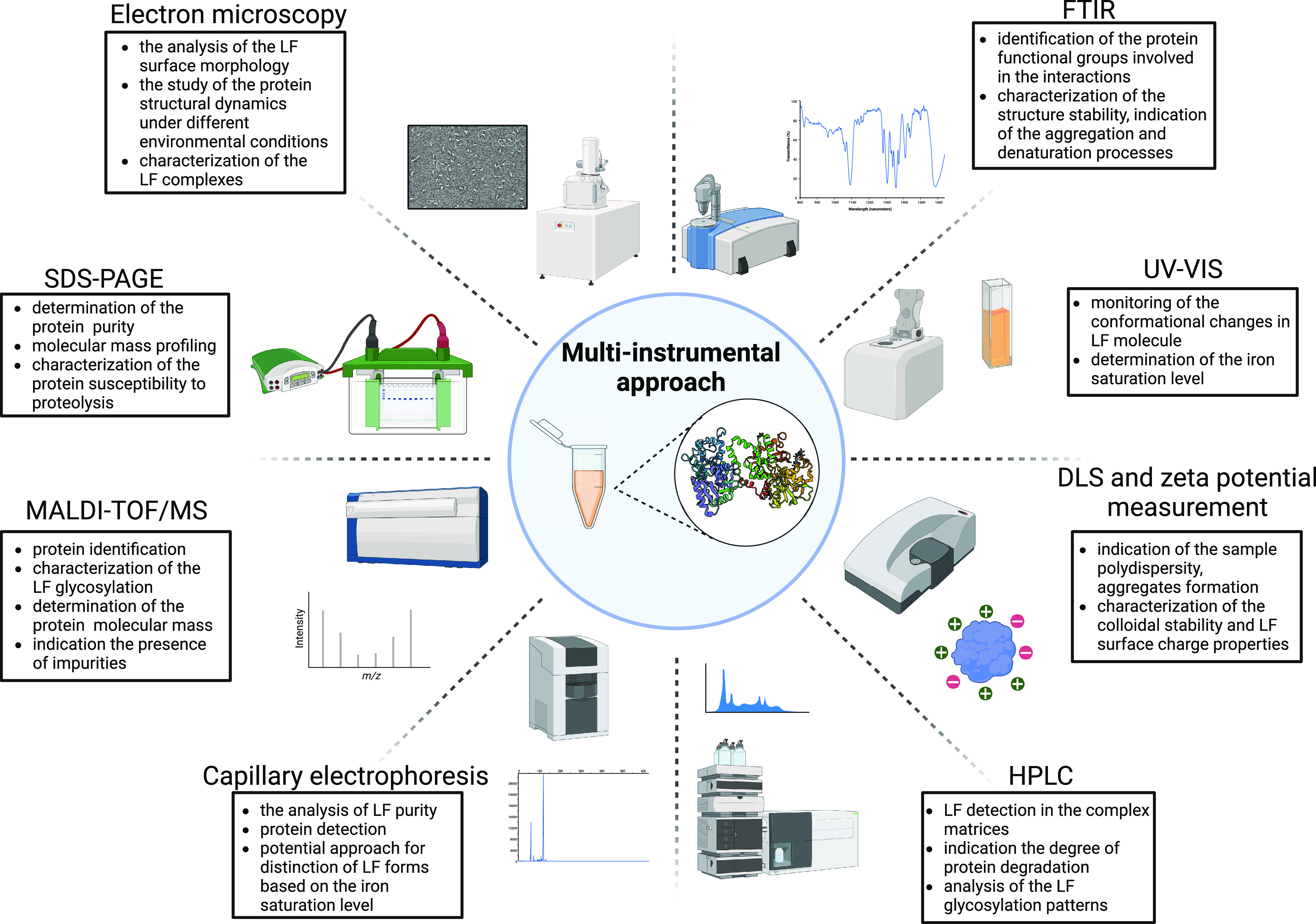
Multi-instrumental approach for studying lactoferrin protein. Created with BioRender.com.

The application of a series of instrumental techniques enables the comprehensive characterization of native LF as well as its complexes, including the molecular mass, isoelectric point, metal saturation level, unfolding degree, secondary structure composition, and conformational stability (Table 2). Besides, the combination of the appropriate analytical techniques will provide the information on the most unknown aspect of LF structure—glycosylation. To the most relevant tools commonly used in the context of LF research are included spectroscopic, electromigration, crystallographic, chromatographic, microscopic, calorimetric, and spectrometric techniques (Figure 3). The advanced instrumental studies on lactoferrin are necessary for the establishment of the relationship between protein structure and its functionality. The present section aims to display the specific details of the methods and the possible issues and challenges during LF detection.

**Table 2:** Specification of the Relevant Instrumental Techniques for LF Characterization

protein characteristic	instrumental technique	classification of the instrumental technique	specific details	applied conditions for LF detection	ref
molecular mass	SDS-PAGE	electromigration	The key principle of the separation relies on the different mobility of the charged particles under an electric field that appear as a result of the difference in their molecular sizes. The molecular mass of bLF varies in the range 78–91 kDa in dependence of the source of isolation and glycosylation state.	protein separation in 4–12% polyacrylamide gel; reducing agent, 2-mercaptoethanol; running buffer, 1× MES; voltage, 200 V; time of separation, 22 min; gel staining, Coomassie Blue R-350	(11, 131)
	MALDI-TOF/MS	spectrometric	MALDI is a soft ionization technique. The appropriate chemical substance is the matrix, which mediates in the energy transfer from laser beam to analyte and then enables its desorption and ionization. LF mass was found at about 82–84 kDa in dependence on the stage of lactation.	MALDI-TOF/TOF mass spectrometer UltrafleXtreme (Bruker Daltonics); laser, Nd:YAG (355 nm); laser frequency, 2 Hz; matrix solution, sinapic acid dissolved in 30:70 (v/v) mixture of acetonitrile and 0.1% trifluoroacetic acid (TFA)—TA30; *m*/*z* range, 10–100 kDa	(13, 130)
iron saturation level	UV–vis	spectroscopic	The total LF concentration is recorded at 280 nm. The characteristic band at 465 nm indicates the iron(III) coordination by LF. The calculation of absorbance ratio *A*_465_/*A*_280_ is relevant for determination of the iron saturation level of LF.	UV300 spectrophotometer (Thermo Scientific, USA); path length of quartz cuvette, 1 cm	(153)
	ICP-MS	spectrometric	In contrast to UV–vis, ICP-MS analysis is considered more accurate for determination of the negligible amount of bounded iron in LF (<2%).	samples of LF in mineralized HNO_3_ analyzed by ICP-MS ELAN spectrometer (PerkinElmer, USA); plasma gas, Ar; gas flow, 15 L/min	(57)
particle charge properties	zeta potential measurements	spectroscopic	Electrophoretic mobility was determined based on the Henry equation. The zeta potential was calculated according to the Smoluchowski approximation. The measured zeta potential is highly influenced by the type of electrolyte (NaCl, KCl) used and ionic strength.	Malvern Nano-ZS ZetaSizer (Malvern Instru ments, U.K.); temperature, 25 °C; cuvette, DTS1070; equilibration time, 2 min	(129, 154)
	IEF	electromigration	Different pI values determined by IEF also might be a matter of the selected separation conditions, e.g. the type of carrier ampholyte (CA), pI marker, buffer composition, and separation time. The possibility of distinction of several LF fractions is based on their pI values.	Multiphor II apparatus (Amersham, Freiburg, Germany); precast gels Servalyt and Blank precotes (Serva, Germany); gel parameters, 126 × 125 × 0.3 mm; temperature, 5–8 °C; voltage, 2000 V; current, 6 mA; power, 12 W	(59, 83)
three-dimensional molecular structure	XRD	crystallographic	XRD provides a detailed insight into the protein’s three-dimensional structure. It indicate the possible metal-binding sites and their geometry, and it is essential for understanding the structural difference between apo- and holo-LF. XRD enables the verification of bLF stability under various environmental conditions. The technique is considered a basis of drug design and development.	synchrotron beamline BM14 at European Synchrotron Radiation Facility (Grenoble, France); space group, *P*2_1_; cell dimensions, *a* = 75.8 Å, *b* = 49.4 Å, *c* = 97.9 Å; resolution range, 2.42–38.7 Å	(46, 60)
secondary structure	CD	spectroscopic	The assignments of individual secondary structures are found in the following ranges: (1) 190–195 nm (max), 208 nm (min), and 220–222 nm (min) for α-helix; 195–200 nm (min) and 215–218 nm (max) for β-sheet. The monitoring of elipticity at 220 nm is frequently to estimate the protein denaturation.	spectropolarimeter J-720 (JASCO, Tokyo, Japan); path length of quartz cell, 10 mm; UV region, 190–260 nm; scan speed, 50 nm/min; temperature, 25 °C	(155)
	FTIR spectroscopy	spectroscopic	Amide I is considered the most relevant for secondary structure profiling: 1660–1650 cm^–1^ (α-helix), 1650–1640 cm^–1^ (random coils), 1680–1670 cm^–1^ (β-turn), 1690–1680 cm^–1^ and 1640–1630 cm^–1^ (β-sheet). Different deconvolution approaches, including Gaussian and Lorentzian fitting, second derivatives with appropriate software program (OMNIC, OriginPro, PeakFit) are frequently used in calculations of the contributions of individual structures in proteins.	FTIR spectrometer (NEXUS, Nicolet, USA) in attenuated total reflectance (ATR) mode; spectral range, 4000–650 cm^–1^	(116, 125)
	Raman spectroscopy	spectroscopic	The region of 1700–1620 cm^–1^ was selected for distinction of each type of secondary structure. In contrast to the FTIR technique, Raman spectroscopy is more sensitive to samples containing nonpolar functional groups and is less influenced by interferences caused by hydrogen bonding between analyte and water molecules.	JASCO NRS-2000C; excitation, Ar^+^ laser (514 nm); detector, charge-coupled device (160 K); spectral resolution, 4 cm^–1^	(71)
changes in the tertiary structure	fluorescence spectroscopy	spectroscopic	Aromatic amino acids constitute about 9% of the total sequence in the bLF polypeptide chain. The measurement of the fluorescence intensity ratio *F*_330_/*F*_350_ is related to protein destabilization, unfolding, and the exposure of aromatic amino acids to a polar microenvironment.	Cary Eclipse spectrofluorimeter (Varian, Middelburg, The Netherlands); emission range, 300–400 nm; path length of quartz cuvette, 1 cm	(73, 156)
aggregation state	DLS	spectroscopic	Destabilization processes such as protein unfolding and formation of larger aggregates accompany a considerable increase of single particle size up to 30 nm. The protein hydrodynamic size was calculated according to the Stokes–Einstein equation.	Malvern Nano-ZS ZetaSizer (Malvern Instru ments, U.K.); temperature, 25 °C; backscatter angle, 173°; cuvette, rectangular polystyrene cell (10 mm path length); equilibration time, 2 min	(72, 157)
	SEC	chromatographic	SEC is the appropriate technique for the evaluation of sample purity. The appearance of the additional fractions might indicate the tendency of LF to self-associate or the ability to form complexes with other milk proteins.	SEC-HPLC system (Agilent Technologies, USA); detector, diode array detector; column, TSKgel G3000SW_XL_ (7.8 × 300 mm, 5 μm, Tosoh Biosciences LLC, USA); flow rate, 0.5 mL/min; temperature, 22 °C; elution type, isocratic; mobile phase composition, 30% (v/v) acetonitrile and 0.1% (v/v)	(72)
	TEM	microscopic	TEM determines the morphologies of protein aggregates (amyloid fibrils, amorphous aggregates). It provides the insight into the mechanism of protein aggregation.	JEOL JEM 1010 electron microscope (Tokyo, Japan); accelerating voltage, 80 keV; sample placement, Formvar carbon-coated nickel grid (200 mesh)	(64)
glycosylation patterns	MALDI-TOF/MS	spectrometric	The protein sequence analysis as well as its structural modifications might be determined based on the characteristic peptide mass fingerprint (PMF) signal list obtained by the appropriate software (Mascot, Sequest). MALDI tends to generate singly charged ions and better tolerates salts and other impurities. The amount of *N*-glycans was explored by deglycosylation.	MALDI-TOF/TOF MS spectrometer (Bruker Daltoniks GmbH, Germany); laser, nitrogen (337 nm); matrix solution, 2,5-dihydroxybenzoic acid (DHB) (10 mg/mL) in 50:50 (v/v) mixture of acetonitrile and water for glycopeptide analysis; mode, reflector positive ion (neutral *N*-glycans), linear positive ion (glycopeptyde), linear negative ion (sialated *N*-glycans)	(158)
	LC–ESI-MS	chromatography coupled with mass spectrometry	The liquid samples subjected to the ESI process aimed to generate gas phase ions typically undergo ionization under high voltage in the metal capillary which is followed by solvent evaporation. ESI favors the formation of large hydrophobic peptides with charge states ranging from +2 to +4, providing more detailed fragmentation patterns. A salted sample might provide evident signal suppression. ESI-MS characterizes by exceptional sensitivity to slight impurities, for example salts, that is expressed by a higher tendency of formation of charged adducts which might also suppress the signal intensity from analyte.	HPLC system (Datasystem Millennium, HPLC pumps Waters 510, detector Waters 486); column, C18 Vydac (250 × 10 mm, 5 μm); gradient elution, 0.01% TFA (solvent A) and 0.07% TFA in 95% acetonitrile (solvent B); flow rate, 3.5 mL/min; single quadrupole mass spectrometer (Micromass); scanning range (*m*/*z*), 300–1600	(151)
thermal stability	DSC	calorimetric	The significant difference in thermal resistance of LF is in dependence on iron saturation. The different ratio of apo- and holo-LFs is capable of modifying the thermal stability of native protein.	device, differential scanning calorimetry (DSC1 STARe System, METTLER TOLEDO, Schwerzenbach, Switzerland); heating range, 25–100 °C; scanning rate, 10 °C/min (nitrogen flow)	(119)
LF–ligand interactions	ITC	calorimetric	ITC is used for the investigation of the mechanism of interactions (affinity, stoichiometry, thermodynamics) between LF and other molecules, especially metals. Based on the determined thermodynamic parameters, the character of LF–ligand interactions (electrostatic, hydrophobic, hydrogen bonding, van der Waals forces) is evaluated.	VPITC microcalorimeter (MicroCal VP-ITC, Malvern Panalytical, Malvern, U.K.); sample cell, LF (1.425 mL); reference cell, MES buffer (pH 5.5); total number of injections, 58 (5 μL each); injection timing, 10 s; interval, 200 s	(157, 159)

### 8.1 Spectroscopic Techniques

#### 8.1.1 Ultraviolet–Visible (UV–vis) Spectroscopy

UV–vis spectroscopy is known as one of the most popular methods for studying the iron-binding properties of lactoferrin. The measurements of the absorbance relationship *A*_465_/*A*_280_ can be helpful in the distinction of the degree of iron saturation in bLF.^153^ Generally, the decrease of the absorption intensity ratio indicates the release of ferric ions from the complex. Remarkably, different glycosylation levels of hLF do not contribute significant changes in UV–vis spectra.^160^ The analogical mechanism of cation−π interactions induced the rise of the absorption peak in the case of lactoferrin complexes with copper(II), manganese(III), and cobalt(III) ions at the following wavelengths: Cu–LF 438 nm (ε = 4800 mol^–1^ L cm^–1^), Mn–LF 435 nm (ε = 9620 mol^–1^ L cm^–1^), and Co–LF 405 nm (ε = 10 340 mol^–1^ L cm^–1^).^161,162^ Remarkably, that characteristic LMCT band was not present after the addition of Mn(II) and Co(II) ions, which might be related to the coordination of these metals to other binding sites in the LF molecule, not including deprotonated tyrosine residues.^160^ Lucas studied protein spectral properties in the presence of Li^+^, Na^+^, and Cs^+^ ions. The authors reported that the change of the absorption peak position was mainly influenced by hydrogen interactions and proton transfer between metal cations and the tyrosyl group.^163^ Indeed, UV–vis spectroscopy is characterized by the much lower sensitivity of iron quantification in comparison to the ICP-OES and ICP-MS techniques. Bokkhim et al. reported that the absorption peak at 465 nm was not visible during the detection of apo-LF.^164^ Despite this, the application of the UV–vis approach is limited in the case of supersaturated LF complexes. According to a basic assumption, holo-LF is capable of binding a maximum of two ferric ions (*q*_m_ 1.4 mg/g); however, Pryshchepa et al. found the 54 total iron-binding sites (*q*_m_ 36.3 mg/g) in one bLF molecule.^165^

#### 8.1.2 Fluorescence Spectroscopy

Fluorescence spectroscopy allows the determination of the stability of LF conformation and indicates unfolding processes. Destabilization effects related to protein interactions with metals might be confirmed by the red shift of fluorescence emission maxima from 330–340 nm to 350–355 nm.^166^ The pH-induced exposure of aromatic amino acids is commonly corresponding to the denaturation of the polypeptide chain. For example, the emission maxima of apo- and holo-LFs at pH 2 were detected at 353 and 348 nm, respectively.^56^ Measurements of the fluorescence intensity ratio *F*_330_/*F*_350_ allow indication of the degree of protein unfolding upon different physicochemical conditions.^73^ For instance, the intrinsic fluorescence might be helpful for monitoring the conformational alterations in the protein structure during iron chelation. As it was previously reported, the level of metal saturation is another factor which affects the position of the LF emission peak. Thus, Wang et al. noticed the characteristic band of apo-LF at 336 nm, while in the case of holo-LF it was shifted to 343 nm, indicating the exposure of tryptophan to a more polar environment.^156^ Similar results were reported previously by Sreedhara et al. that indicated the maximum emissions at 337.07 and 342.96 nm, for apo-LF and holo-LF, respectively.^56^ Remarkably, Moshtaghie et al. did not observe any difference in the position of the emission band (λ_em_ 355 nm) between apo- and holo-LFs; nevertheless, the fluorescence intensity decreased (quenched) to 35% after the addition of metal.^167^ The presence of metals has a tendency to reduce the fluorescence (quenching) of the LF molecule. Previously, Harrington et al. related the fluorescence quenching induced by Fe(III), Mn(III), and Co(III) ions to intermolecular energy transfer which corresponds with specific interactions of metal ions with tyrosine residues and conformational changes around LF binding sites.^132^ Ainscough et al. showed that among all of cations, ferric ions were the most effective quenchers, probably because of their the highest binding capacity to LF.^168^ The analysis of the protein fluorescence quenching is made with the Stern–Volmer relationship, which is expressed by eq 1:
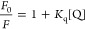
1where *F*_0_ is the protein fluorescence intensity in the absence of quencher, *F* is the protein fluorescence after the addition of quencher, *K*_q_ is the Stern–Volmer quenching constant [M^–1^], and [Q] is the molar concentration of the quencher [M].

The mentioned model is capable of delivering the essential information on the binding affinity and stoichiometry of complex formation. The major problem that arises in studying LF conformational changes upon iron saturation is based on the nonlinear behavior of the Stern–Volmer plot.^168^ This phenomenon might be explained by the accessibility of fluorophores located in two potential binding sites and their different abilities to interact with metal ions.^169^ Therefore, the results obtained by spectrofluorimetry seem to be complicated for interpretation of the mechanism of Fe–LF complex formation. In this case, experimental conditions, e.g. temperature, solvent type, pH, and concentrations of protein and ligand, should be carefully controlled, since all of these factors might affect the plot linearity.

#### 8.1.3 Fourier-Transform Infrared (FTIR) Spectroscopy

FTIR spectroscopy is an essential instrumental tool which delivers necessary information on the stability of protein conformation and ensures the identification of active functional groups involved in protein–metal binding. Positions of characteristic amide bands at the spectral ranges of 1700–1600 cm^–1^ (amide I), 1600–1500 cm^–1^ (amide II), and 1350–1200 cm^–1^ (amide III) allow distinguishing the type of secondary structure (α-helix, β-sheet, turn, loop, random coil, unordered structure) and determining the strength of interchain hydrogen bonding.^170^ Asp and Glu residues are commonly found in the ranges of 1430–1380 cm^–1^ and 1600–1550 cm^–1^ (amide II), respectively. The frequency of these peaks might be considerably shifted upon interactions with metal ions.^171^ The difference Δ(ν_a_ – ν_s_) indicates the form of metal chelation by carboxylate (unidentate, >260 cm^–1^; bridging, <200 cm^–1^; pseudobridging, <150 cm^–1^; bidentate, <105 cm^–1^).^172^ These calculations might be an helpful in verifying the geometry of LF binding sites upon metal binding. The combination of FTIR spectroscopy with thermoanalytical techniques, especially DSC, might provide necessary information on the stability of the LF tertiary structure. Recently it was discovered that absorption peaks at 1690 and 1615 cm^–1^ frequently appear upon protein denaturation, while the band at 1627 cm^–1^ reflects the formation of amyloid aggregates.^64,116^ FTIR spectroscopic analysis study of the protein–ligand molecular interactions might provide the important information on the iron role in the LF conformational stability. For example, FTIR spectrum obtained by Pryshchepa et al. confirmed the contribution of tyrosine residues and deprotonated aspartate and glutamate side chains in the chelation of ferric ions.^165^ What is important is that the performed deconvolution analysis by Hadden et al. did not detect any visible difference in the secondary structure content of LF after the removal of iron.^116^ So it was in later research.^173^ Thus, it is worthwhile to conclude that FTIR spectroscopic analysis might not be a suitable approach for distinction of LF forms at the different saturation states.

#### 8.1.4 Raman Spectroscopy

Raman spectroscopy is a fundamental instrumental technique which is relevant for the study of structural stability and detection of inter- and intramolecular processes within a molecule such as folding, defolding, and aggregation.^174^ In addition, it enables monitoring of the conformation changes in the protein structure upon modification with different ligands. Similarly, as in the case of FTIR spectroscopy, Raman spectroscopy might be combined with DSC studies to monitor the heat-induced changes in the secondary structures of apo- and holo-LFs. As a result, Iafisco et al. has pointed to a positive correlation between the intensity of bands assigned to tyrosine at 1520 and 1170 cm^–1^ and the metal saturation level.^71^ In contrast, the study on iron-binding proteins (ferritin, transferrin) indicated the appearance of new peaks related to Fe–O and Fe–NO coordination bonds in the range 460–420 cm^–1^.^175^ The direct application of Raman spectroscopy in the study of protein glycosylation might be challenging due to possible signal overlapping that causes some difficulties in spectra interpretation. Previously, it was found that LF deglycosylation is typically accompanied by visible conformational changes and polarity modifications, especially around aromatic amino acids. Consequently, the band shifts might be observed in a few spectral regions: 1610–1600 cm^–1^ (Tyr), 1360–1340 cm^–1^ (Trp), 1280–1210 cm^–1^ (Tyr), 1010–1000 cm^–1^ (Phe), and 880–870 cm^–1^ (Trp). Unfortunately, the glycosylation profiles in bLF were poorly studied with Raman spectroscopy until this time. Ainscough et al. studied the spectral properties of metal-saturated hLF complexes substituted with Fe(III), Cu(II), Mn(III), Co(III), and Cr(III) ions.^168^ On the basis of the obtained Raman spectra, the authors assumed similar binding sites for all of the synthesized complexes. This indicated the presence of intensive signals attributed to tyrosine residues in the frequency ranges 1660–1650 cm^–1^, 1510–1490 cm^–1^, 1280–1250 cm^–1^, and 1170–1160 cm^–1^, which confirmed their strong coordination with metals. One of the meaningful disadvantages of Raman spectroscopy is related to fluorescence interferences that are induced by the operating laser excitation wavelength (mostly 532 nm).^176^ As a result, it leads to signal suppression and the lowering of the method sensitivity. Surface enhanced Raman spectroscopy (SERS) is an advanced approach of Raman spectroscopy in which the analyte excitation is assisted by metal nanostructures (AgNPs, AuNPs). In contrast to traditional Raman spectroscopy, it characterizes by improved sensitivity and ensures the reduction of the fluorescence background. Despite this, SERS analysis might demonstrate a low reproducibility due to size heterogeneity of nanoparticles. In addition, it was revealed that the LF concentration had an impact on the spectral characteristics; thus the application of the SERS approach requires a detailed optimization of the conditions.^177^

#### 8.1.5 Dynamic Light Scattering (DLS) and Zeta Potential Measurements

DLS is a relatively simple technique which allows for rapid determination of the hydrodynamic diameter of dispersed biomolecules. The size of LF particles is relatively small and ranges between 2 and 13 nm.^63^ DLS is considered an ideal technique for monitoring the sample polydispersity, as it is known that LF not only occurs in monomeric form but also might undergo self-association. As it was reported, the formation of LF aggregates was regulated by intramolecular electrostatic interactions, thereby forming a spherical micelle structure. What is interesting is that the direct role of chelated iron in the LF hydrodynamic size has not been extensively studied. According to crystallographic studies apo-LF showed a more open structure, while holo-LF was characterized by a more compact conformation.^47^ Thus, it might be assumed that the protein hydrodynamic size will decrease as the iron saturation increases. Valiño et al.^129^ reported that the diameter of apo-LF varied from 8 to 13 nm in the pH range 4–9. Unfortunately, information on the size changes of the saturated LF form is limited. Jabeen showed that the hydrodynamic diameter of the diferric LF dissolved in Tris buffer at pH 8 was equal to 6.94 nm.^60^ In addition, the determined size of the iron-saturated C-lobe was 3.83 nm (pH 6.5), while the same fragment complexed with zinc ions was about 4.21 nm (pH 3.8). It is worth noting that the values of hydrodynamic size of apo- and holo-LFs could be barely comparable, since the measurements should repeated at the same conditions. The results might differ in dependence on the pH, the temperature, the protein concentration, the type of used electrolyte, and the ionic strength. Remarkably, Mela et al. showed that the salt-induced aggregation with 100 mM NaCl solution promoted the growth of the LF diameter even to 100 nm.^63^ Besides, protein functionalization with other ligands frequently leads to modification of the lactoferrin surface and structural rearrangements. The typical increase of LF hydrodynamic diameter might be related to the reduction of conformational rigidity as well as to the formation of additional coating layers.

Interestingly, the way of protein drying also has a valuable impact on the particle size.^107^ The measurement of the electrokinetic potential (zeta potential, ζ) is an essential step in the designation of protein surface electrical properties and colloidal stability. Values higher than ζ ± 25 mV inform about the prevalence of electrostatic repulsions over attraction forces in analyzed dispersions.^178,179^ Pryshchepa et al. showed that the LF charge is highly dependent on sample conditions, and values of ζ gradually changed from +20 to −6 mV over the pH range 4–9.^180^ Previous results indicate that the LF surface remains positively charged over the wide pH range. The reduction of the zeta potential to 0 mV occurs around pHs close to the protein isoelectric point. According to the majority of works, it was revealed that the LF isoelectric point is 8–9, which implies the prevalence of basic-character amino acids in the protein sequence. On the contrary, Yoshida reported that the LF surface charge was less positive and the pI value was found around 4.8–5.3.^181^ As it was previously mentioned, the pI of bLF is highly dependent on the iron saturation level. Significant conformational modifications induced by iron saturation provide valid changes in the bioavailability of amino acids, especially with charged side chains. According to Bokkhim et al., the LF desaturation accompanies the exposure of negatively charged regions; thereby the pI was indicated at about 6.3.^119^ In contrast, the ionizable protein groups tend to be bulked inside the compact structure of holo-LF; thus the determined pI increased to 8.6. Besides, the negatively charged surface might be neutralized as a result of interactions with ferric ions. The measurement of the zeta potential is widely used for characterization of the colloidal stability and surface charge properties of LF, but this approach is not relevant for the quantitative analysis of the iron content. As in the DLS studies, the obtained zeta potential results are strongly affected by the measurement conditions. Functionalization of proteins usually accompanies evident changes of their physicochemical features. Immobilized metal ions tend to electrostatically interact with protein functional groups (in particular, with Asp and Glu) inducing neutralization of the surface charge or inversion to more positive.^182^ Besides, the study of AgNPs coated by bLF at pH 7 showed that the protein has a tendency to reduce the negative surface charge of metal nanoparticles from −28 to −6 mV.^183^ Destabilization of AgNPs was predominantly induced by their interactions with basic side chains of bLF. The opposite effect was observed in LF complexes with hyaluronic acid (HA).^184^ As a result, the zeta potential of pure LF has changed from +9.6 to −30 mV in LF–HA systems.

### 8.2 Electromigration Techniques

#### 8.2.1 Sodium Dodecyl Sulfate Polyacrylamide Gel Electrophoresis (SDS-PAGE)

SDS-PAGE analysis has been widely used for the verification of molecular profiles in biomolecules. The key principle of the separation relies on the different mobilities of charged particles under an electric field that appear as a result of the difference of their molecular sizes (2):
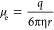
2where μ_e_ is the particle electrophoretic mobility [m^2^ s^–1^ V^–1^]; *q* is the particle charge [C]; η is the dynamic viscosity of the medium [Pa·s]; *r* is the size of the particle [m].

A single bLF band is traditionally expected in the range 78–80 kDa; nevertheless, small portions of high molecular wieght aggregates also might be present. What is important is to select the proper LF concentration since the small protein amount might undetectable on the electropherogram. Gel permeation chromatography (GPC) might be alternatively utilized instead of SDS-PAGE. In this approach, the molecules are separated based on their hydrodynamic sizes. The basic principle of GPC fractionation assumes that the larger molecules are eluted first from the column, while the smaller components tend to interact with column porous beads; thus they are eluted last. GPC is considered a relevant technique for the detection of high molecular weight (HMW) LF associates. As it was mentioned previously, LF tends to interact with other whey proteins and also could undergo self-aggregation. LF tetramers belong to one of the most abundant multimeric fractions whose bands might be observed at about 300–350 kDa.^185^ Wang et al. indicated several LF fractions at about 150, 250, 300, and 800 kDa as a result of interactions with other milk components, e.g. caseins, immunoglobulins, serum albumin, and lysozyme.^14^ The LF size might be affected by various factors, e.g. degree of glycosylation, source of isolation, way of storage, and type of bounded ligand. It has been reported that the molecular weight of unsaturated hLF after the complete removal of carbohydrate chains decreased from 80 to 75 kDa.^186^ Generally, it is expected that the molecular weight of LF is not strongly affected by the degree of metal saturation. Nevertheless, Pryshchepa et al. observed a slight difference in mobility between native bLF and supersaturated Fe–bLF.^187^ Nagasako et al. explained this phenomenon by the presence of additional metal binding sites which contribute to the modification of the protein surface charge.^52^ Ying et al. confirmed the difference in electrophoretic mobility between both saturated and unsaturated forms of LF.^188^

Iron saturation is accompanied by visible conformational changes in the LF structure; however, the authors pointed the impossibility of the distinction of mono- and diferric LF forms by SDS-PAGE analysis. In addition, it was found that iron chelation first occurs in the N-lobe and then in the C-lobe. SDS-PAGE analysis is widely applied during enzymatic digestion in order to estimate the susceptibility of protein structure under different proteolytic conditions. Sharma et al. determined that the size of the bLF C-lobe generated by hydrolysis with proteinase K was slightly bigger than the N-fragment, as their bands appeared at 38.6 and 38.4 kDa, respectively.^58^ Rastogi et al. also did not notice any significant difference in the size between of both lobes of bLF obtained by tryptic digestion, where each was at about 38 kDa.^46^ The gradual dropping of pH to 2–3 creates more favorable conditions for the acidic hydrolysis of LF and provides cleavage of the polypeptide chain and formation of smaller biologically active protein fragments.^189^ On the basis of *in vivo* studies Furlund et al. observed the greatest degradation level in the LF molecule after treatment with gastrointestinal juice (pH 2.5) after 30 min.^190^ Besides, the stability of LF might vary depending on the protein origin. According to Ma et al.’s studies, it was detected that bLF exhibited much lower resistance to pepsin digestion in comparison to hLF.^191^ The difference may be a result of glycosylation heterogeneity in proteins from other species. SDS-PAGE is an essential tool used in LF detection and the examination of its purity in the individual fractions during the isolation process. For example, α-lactalbumin as one of the most abundant milk proteins is commonly observed at 15 kDa on LF electropherograms.^72^ Therefore, the presence of the additional patterns will indicate the low purity grade of the achieved product as a result of inappropriate separation conditions.

#### 8.2.2 Capillary Electrophoresis (CE)

Capillary electrophoresis is regarded as a more economically friendly separation method in comparison to chromatographic techniques. Similarly to SDS-PAGE, the molecule separation is based on the difference in electrophoretic mobilities. The major problem that arises in CE analysis is during the protein detection related to interactions between analyte and coated functional groups of the capillary wall, inducing the electroosmotic flow (EOF) problem. As it was shown, the LF detection might be performed in positive voltage (pH 4) and in negative voltage (pH 10) as well. Nevertheless, it was confirmed that the LF analysis at pH 10 provided the improved sensitivity and the peak shape.^192^ Accordingly, it could be assumed that the optimal analysis of LF molecules by CE is more favorable at the basic conditions above the determined isoelectric point, since the protein as well as silanol groups of the capillary wall both remain negatively charged. Recently, Li et al. performed an effective separation of LF by modification of running buffer with the nonionic surfactant polyethylene glycol dodecylether (Brij 35) using acetic acid as the sample buffer.^193^ It was observed that the addition of Brij 35 had a positive effect on the specificity and selectivity of the analysis in the context of LF study. Affinity capillary electrophoresis (ACE) is one of the developed approaches of the traditional CE which is based on the addition of some modifiers to running buffer which characterize a high binding affinity to analyte and aim to improve the separation efficiency. Consequently, the formed protein–ligand complex minimizes the risk of interactions with the capillary wall and reduces EOF. For example, Heegaard et al. showed that no peak of LF was observed by using unmodified sodium phosphate buffer (pH 6–8); however, LF was detectable after the addition of heparin to the buffer solution (pH 8).^194^ The identification of apo- and holo-LF forms by CE was not comprehensively investigated until now. Nowak et al. studied the separation of apo- and holo-LFs testing fused-silica and neutral capillaries.^195^ The results did not show significant changes in the migration time of both forms; nevertheless, their peak positions were exchanged after the inversion of polarity in the capillary. Thus, it might be assumed that the CE approach is not selective for proteins with different iron saturation degrees since the electrophoretic mobilities of apo- and holo-LFs do not differ significantly between each other.

### 8.3 Crystallographic Studies

#### 8.3.1 X-ray Diffraction Analysis (XRD)

Crystallographic analysis provides detailed information about the organization of the protein structure on the atomic level. Moreover, the visualization based on electron density maps is a powerful tool for monitoring conformational changes in complex-organized structures, like metalloproteins. It gives an opportunity to determine the number of potential binding sites, their geometry, localization inside crystallized biomolecule, and coordination distances.^196^ Shongwe et al. reported the geometric rearrangements in the N-lobe of hLF upon iron(III) substitution by copper(II) ions.^197^ According to the gained results, incorporation of Cu^2+^ ions was accompanied by the formation of a monodentate complex (instead of the observed bidentate carbonate site, as in Fe–LF) with carbonate anions which led to the transition of the coordination geometry from octahedral to square pyramidal. Indeed, XRD studies clarify the nature of protein–metal interactions and predict the biological properties of created complexes. Smith et al. mentioned the importance of interactions between ruthenium(III) and hLF in the development of novel antitumor agents.^198^ Crystallographic research proved the octahedral geometry of the Ru–LF complex was maintained by two histidine residues and four water molecules.^199^

### 8.4 Chromatographic Techniques

#### 8.4.1 High-Performance Liquid Chromatography (HPLC)

High-performance liquid chromatography (HPLC) is known as a fundamental analytical tool widely employed for sensitive detection and accurate quantification of biologically active components in the complex matrix. In the case of LF analysis, an extensive pretreatment procedure of milk samples is required. It normally obligates defatting, the precipitation of caseins, and centrifugation.^200^ It is worth noting that HPLC is relevant for the quantification of intact LF but not protein hydrolysates.^201^ HPLC is applied during the optimization of protein isolation to determine the method’s effectiveness, the denaturation level, and the content of biologically active LF. Parra-Saavedra et al. successfully performed a separation and determination of LF concentration in human milk.^202^ The authors selected Kinetex XB C18 as a stationary phase and subjected a sample to gradient elution with a mixture of A and B phases as 0.1% (v/v) trifluoroacetic acid in water and acetonitrile, respectively. On the other hand, Zhang et al.^203^ reported that C4 was more optimal for bLF quantification in contrast to a C18 column. The authors pointed out that the protein separation by a column with a shorter carbon chain (less hydrophobic) improved the overall peak shape, reduced the baseline drift, and provided a better repeatability.^203^ Despite the high efficiency and short time of analysis, the separation by C4 columns might induce worse peak resolution in the case of complex samples. Remarkably, a C8 column might be an alternative variant instead of C4 and C18. The LF concentration in the goat milk was quantified using a Poroshell 300SB-C8 column and linear gradient elution with water/acetonitrile/trifluoroacetic acid at the following volume ratios (v/v) of phase A (95:5:0.1) and phase B (5:95:0.1).^204^ Tsakali et al. applied similar conditions for the determination of bLF content in feta cheese whey utilizing a Zorbax SB 300-C8 column and gradient elution with an acetonitrile, water, and trifluoroacetic acid mixture at the proportions of 50:950:1 v/v (solvent A) and 950:50:1 v/v (solvent B).^205^ While HPLC is considered a great technique for the analysis of native proteins, liquid chromatography coupled with tandem mass spectrometry (LC–MS/MS) is suitable for overall bLF quantification as well as for the identification of characteristic peptides. The determination of bLF in complex samples is typically performed in multiple reaction monitoring (MRM) mode. The major task during the MRM approach is based on the selection of the appropriate precursor ion and then to optimize the collision energy. For example, Yuan et al. chose the ETTVFENLPEK (230–240) fragment for further investigations due to the highest mass response among other peptides.^206^ As it was described in an earlier section, the major advantages of the LC–MS/MS approach is related to a capability of monitoring protein post-translational modifications. In addition, LC–MS/MS was also utilized for evaluation of the effect of protease action on the level of bLF degradation. The peptide separation was performed using a C18 column as the stationary phase and a mixture of 70% acetonitrile and 0.1% formic acid (v/v) as the mobile phase. The number of identified peptides during *in vitro* and *in vivo* conditions was variable and depended on a number of factors, e.g. pH, time, and protease composition (gastric and duodenal juices).^190^

### 8.5 Electron Microscopy Techniques

Electron microscopy is considered a powerful tool which is frequently applied in the estimation of the quality of milk products by characterization of their microstructures.^207^ Imaging techniques also are suitable for an effective monitoring of structural changes in samples under different environmental factors, e.g. temperature, pH, and ionic strength, and the detection of precipitates. Rodzik et al. have noticed apparent changes in the morphology of individual caseins upon their modification with Zn^2+^ ions.^208^ It has been reported that the formation of protein aggregates was preferred at the highest metal concentration (600 mg/L). Imaging techniques are important in verifying the decomposition heterogeneity of adsorbed metal. In addition, metal immobilization onto the protein surface represents a novel approach of nanocomposite synthesis, which in turn corresponds with expanded biological activity of biomolecules. Obtained micrographs by Król-Górniak et al. demonstrated that incorporation of zinc ions into the ovalbumin structure was followed by the formation of ZnO nanostructures.^209^

### 8.6 Calorimetric Techniques

#### 8.6.1 Isothermal Titration Calorimetry (ITC)

ITC is a popular nondestructive calorimetric technique widely used for the biophysical study of protein–ligand interactions. Various parameters characterizing the metal binding to the biomolecule, including enthalpic (Δ*H*) and entropic (Δ*S*) changes as well as stoichiometry (*N*), might be determined by ITC analysis. In turn, Δ*H* and Δ*S* values will provide the information concerning the nature of binding forces between the protein and metal ions and indicate the spontaneity (3) of protein–ligand complex formation:^184^
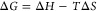
3where the following imply, for Δ*G* < 0, a spontaneous process; for Δ*G* = 0, binding equilibrium; and, for Δ*G* > 0, a nonspontaneous process.

Thus, the following relationships are attributed to (1) for Δ*H* > 0 and Δ*S* > 0, hydrophobic interactions; (2) for *H* < 0 and Δ*S* < 0, hydrogen bonding and van der Waals forces; and (3) for *H* < 0 and Δ*S* > 0, electrostatic interactions.^183^ Greater values of the association constant (*K*_A_) indicate higher stability of the Me–LF complex. Tang et al. reported the highest binding affinity of zinc ions to LF (*K*_A_ = 2.7 × 10^5^ L/mol) in contrast to the rest of whey proteins.^210^ Accordingly, for calculated thermodynamic parameters (Δ*H* = −100 kJ/mol; Δ*S* = −250.5 kJ/mol·K), the prevalence of hydrogen and van der Waals interactions in the Zn–LF binding mechanism can be assumed. In contrast, Bou-Abdallah et al. reported that hTF binding with Fe^3+^, Ti^4+^, VO^2+^, and VO_3_^–^ is a spontaneous process predominantly driven by electrostatic interactions.^211^

#### 8.6.2 Differential Scanning Calorimetry (DSC)

DSC is a popular calorimetric technique which allows determination of thermostability and monitoring of the phase transitions of LF and LF complexes under heating conditions. Protein denaturation is an endothermic process whose value of enthalpy change (Δ*H*_d_) is correlated with the thermal resistance of the biomolecule.^212^ It has been reported below that iron saturation positively influences LF thermal properties. Thermograms obtained by Bokkhim et al. showed that the chelation of two ferric ions per LF molecule corresponded to a significant shift of the denaturation peak (*T*_d_) from 70–71 °C to 90–92 °C.^40^ Remarkably, DSC might potentially be applied for distinction of LF forms based on their iron saturation states. As it was reported, native alpaca LF exhibited three peaks on the thermogram at 66, 77, and 89 °C that are probably related to apo, monoferric, and diferric forms, respectively.^19^ Other authors highlighted that the thermostability of native LFs among the species might vary in dependence of contributions of these LF forms.^125^ In turn, two denaturation peaks observed at 61 and 90 °C for bLF might be explained by the different thermostabilities of the N- and C-lobes and their abilities to release iron.^119,213^ As it was mentioned before, the content of bounded iron in LF is strictly dependent on pH; thus the selected medium conditions also have a considerable effect on the protein stability under heating. The denaturation temperatures of apo- and holo-bLFs dissolved at pH 3 were registered at 36 and 49 °C, respectively.^56^ Nevertheless, they increased to 66 °C (apo-LF) and 90 °C (holo-LF) at pH 7. Importantly, the applied experimental conditions had an impact on the reversibility of LF denaturation. For example, it was proved that LF heating from 5 to 115 °C (at a heating rate of 2 °C/min) provided irreversible changes in the protein structure without any opportunity of conformation recovery upon cooling.^71^ Thus, a DSC study might deliver the essential information on the thermal behavior of a protein and its susceptibility to denaturation, which is especially required for the optimization of an isolation method to receive the biologically active protein.

### 8.7 Spectrometric Techniques

#### 8.7.1 Inductively Coupled Plasma Mass Spectrometry (ICP-MS)

ICP-MS is a powerful analytical tool widely applied for the comprehensive characterization of protein–ligand complex formation. Generally, it provides an accurate determination of the level of metal saturation in proteins.^57^ Besides, the research group headed by Pomastowski has been studying the sorption mechanism between milk proteins and metals ions for a long time by using the ICP-MS technique. The basic principle of such an approach relies on the quantification of unbounded metal in the filtrate. The obtained results might be sufficiently implemented in modeling protein–metal sorption and kinetic isotherms and selecting the most optimal conditions of complex synthesis. The Freundlich and Langmuir models are known as the simplest adsorption isotherm models which are traditionally utilized in the analysis of binding processes. For example, Pryshchepa et al. have determined that LF saturation with ferric ions occurs mostly according to the Freundlich model (*K*_F_ = 4.956 mg/g, 1/*n* = 0.454, *R*^2^ = 0.9994), which implies the heterogeneity of the protein surface and the multilayer character of metal sorption.^187^ On the other hand, the formation of the Ag–LF complex showed more fitting to the Langmuir model (*K*_L_ = 19.94 L/mg, *g*_m_ = 1.55 mg/g, *R*^2^ = 0.971), indicating binding of metal ions as a monolayer on the homogeneous adsorbent.^131^

#### 8.7.2 Matrix-Assisted Laser Desorption/Ionization with Time-of-Flight Analyzer Mass Spectrometry (MALDI-TOF/MS)

MALDI-TOF/MS represents an innovative instrumental approach for the accurate identification of native proteins as well as their peptides. The determined molecular weight of bLF ranges from 78 to 84 kDa and varies in dependence of the presence of metal and the level of protein glycosylation.^131,214^ Pryshchepa et al. has noticed the increase of molecular mass after bLF modification with silver ions.^131^ In addition, MALDI-TOF/MS is a useful tool widely applied for evaluation o fthe purity grade of bLF by detection of the presence of additional biomolecules.^130^ Thus, it was indicated that preliminary ultrafiltration by membranes with a molecular weight cutoff of 50 kDa allowed for separation of bLF from smaller peptides.^180^ MALDI-TOF/MS coupled with SDS-PAGE is an important approach in the determination of bioactive protein fragments resulting in proteolytic digestion. MALDI-TOF/MS analysis performed by Jia et al. sufficiently expressed glycosylation patterns of bLF during a particular lactation period.^13^ According to the obtained spectrometric profile, the authors noted that the transition milk contained the highest total amount of sialic acid in LF compared to colostrum and mature milk. Previous studies have been mentioned sialic acid as an important carbohydrate moiety which might contribute to protein antimicrobial and antiviral activity.^149,151^ These findings might be helpful in verifying the optimal conditions for LF isolation, which will allow obtaining the protein with relevant biological potential.^215^

## 9 Biological Potential of LF Molecule

LF is commonly known as a multifunctional biomolecule which has great potential as an antibacterial, antiviral, immunomodulatory, antioxidant, prebiotic, and anticancer agent. The considerable nutraceutical and therapeutic properties might be successfully realized in biomedical applications.^30,216^ In addition, the recent trend of LF functionalization with other molecules (polyphenols, fatty acids, metal ions) might contribute not only to the improvement of the protein primary features but also to the development of novel complexes with a broader functionality range (Table 3).

**Table 3:** Biological Potential of LF and Related LF Complexes

biological activity	LF form	information	ref
antioxidant	apo-LF	relatively high binding affinity of apo-LF to Fe^2+^ and Cu^2+^ allows reduction of the prooxidant effect of these ions (Fenton reaction)	(217, 218)
	holo-LF	activation of the gene expression of antioxidant markers; overexpression of antioxidant enzymes	(219)
	LF–Se	enhanced activity of antioxidant enzymes (GPx, GR, GST) within cells and tissues with deficiency of selenium; selenium is capable of interacting with glutathione and enables maintaining equilibrium between the oxidant/antioxidant systems	(220)
anticancer	holo-LF	potential activator of natural killer cells inducing apoptosis cancer cells (MDA-MB-231, MCF-7); modulation and decrease the expression of inhibitors of apoptotic proteins (surviving); overexpression of Bcl-2 pro-apoptotic proteins (Bax, Bak), mediated in the mitochondrial pathway of apoptosis	(35, 221)
	CGA–LF	LF complex with chlorogenic acid (CGA) inhibited the proliferation of human colon cancer cells (SW480) in a dose dependent manner (the most optimal mixture consists of 100 μM CGA and 200 μM LF); treatment of cancer cells with CGA–LF complexes promote their apoptosis	(222)
	LF–OA	treatment of cancer cells (HepG2, HT29, MCF) with lactoferrin–oleic acid complexes (LF–OA) induced the activation of mitochondria apoptosis pathway, which corresponded with expression of caspase-3 and pro-apoptic Bax protein; overexpression of p-JKN regulator confirmed the possibility of death receptor-mediated apoptosis pathway	(223)
antibacterial	apo-LF	limitation of the availability of iron to microorganisms resulting in direct binding of this element in the protein structure contributing to host defense against pathogens; antibacterial activity of LF against Gram-positive bacteria (*S. epidermidis*, *B. cereus*) was greater than against Gram-negative (*C. jejuni*, *Salmonella*), which suggests their higher sensitivity to iron deficiency; direct interactions of LF with components of microbial cells with lead to increase the permeability of their membranes resulting in destabilization of structure; prevalence of basic amino acid in LF sequence provides its high binding affinity to negatively charged lipopolysaccharides of bacteria membranes; interaction of LF with LPS caused the inhibition of growth of Gram-negative bacteria resulting in damage to cell; antibacterial activity of LF can be weakened in presence of some cations (Mg^2+^ and Ca^2+^) and anions (HEPES, phosphate, and citrate), which indicates the importance of electrostatic interactions during binding of LF to bacteria surface; citrate is known as a strong chelator for ferric ions which will compete with LF for metal binding and consequently will modify the protein antimicrobial properties; higher antibacterial activity LF peptides (lactoferricin) in comparison to native protein might be related to less branched structure which facilitates its interaction with bacteria cell	(7, 224−228)
	holo-LF	antibacterial effect of LF (0.1–2.0 mg/mL) against *P. aeruginosa* was based on the destructive of biofilm around its cell surface; addition of FeCl_3_ significantly decreased antibiofilm effect of LF, suggesting that free ferric ions may participate in the process of biofilm formation	(229)
	Ag–LF	Ag–LF complex showed germicidal activity against pathogenic bacteria; inhibition of Gram-positive and Gram-negative bacteria (*E. faecalis*, *E. coli*, *P. aeruginosa*, and *S. aureus*) growth was caused by the antibiofilm activity of the complex	(230, 231)
antiviral	bLF	LF is able interact with viral particles and also competes with them for binding to common receptors, avoiding in that way the adsorption and entering of viruses into the cells; neutralization of HCV resulting in rapid interactions of virus with bLF occurred much faster than entering of HCV into the cells; it was reported that sialic acid as part of LF glycan chain is not involved in these interactions, but it was reported that desialylated bLF exhibited a higher antiviral effect against rotavirus compared to native bLF; enhancing of antirotavirus activity after removing of sialic acid is related to facilitation of binding of LF with virus; incorporated ferric ion could contribute to the antiviral activity because holo-LF exerted more effective inhibition of HCV than apo-LF; different activity could be also caused by conformational alterations that occur in LF structure after metal binding; moreover, LF saturated with such metals as Fe^3+^, Mn^2+^, and Zn^2+^ was characterized by higher activity against HIV compared to apo-LF	(126, 151, 232, 233)
	Zn–LF	antiviral activity against poliovirus of Zn–LF complex was directly correlated with degree of saturation of LF; inhibition of viral replication process was based on the binding of LF–metal complex to cell surface and transport of metal ion across cell membrane that led to interference of virus maturation	(234, 235)
	holo-LF	Fe–LF exhibits higher anti-HIV activity in T-cell line than other Mn–LF and Zn–LF complexes; as the authors remarked, preincubation of cells with protein–metal complex induced more effective inhibition of HIV infection; Fe–LF may be applied as potential antiviral agent in some diet supplements	(233)
prebiotic	bLF	prebiotic activity of bovine lactoferrin was compared *in vitro* as well as in fresh cheese samples; it was indicated that bLF promoted the growth of probiotic bacterial strains (*Lactobacillus casei*) only *in vitro*, while their population in fresh cheese samples was not changed probably because of the presence of psychotropic bacteria	(236)
	holo-LF	iron-saturated form of LF stimulated the growth of LAB strains (*Lactobacillus* *delbrueckii* ssp. *bulgaricus*, *Streptococcusthermophilus*) in the yogurt which might be directly related with chelated metal	(237)
	Mn–LF	prebiotic potential of Mn–LF complexes was examined monitoring the number of *Lactobacillus* strains (*L. plantarum* and *L. rhamnosus*); significant population growth was observed after 24 h of incubation of bacterial culture with Mn–LF; the authors related such effect to manganese uptake which contributes probiotic viability	(24)
anti-inflammatory	bLF	LF supplementation reduced the activation of the NF-κB signaling pathway which corresponded with suppressed expression of pro-inflammatory cytokines (TMF-α, IL-1β) in uterine tissue	(238)

The development of advanced LF-based formulations accompanies a number of issues which are mainly related to the appropriate delivery of such biologically active preparations. In order to enhance the LF bioavailability to a human organism simultaneously allowing for the maintenance of the required structural and biological features, several key delivery carriers were proposed.^239^ For instance, LF conjugates with silver nanoparticles (AgNPs) have shown a great antiviral effect against herpes simplex virus type 2 (HSV-2).^178^ The inhibition mechanism might be related to strong interactions between the LF–AgNPs complex and virus, which enabled prevention of the invasion of host cells. In contrast, Abdalla et al. reported that chitosan-stabilized LF–AgNPs composite exhibited a strong antibiofilm activity against pathogenic bacteria (*S. aureus*, *P. aeruginosa*) with no cytotoxic effect on the living cells.^231^ Besides, Softisan based LF nanoemulsion (W/O/W) revealed a great antimicrobial effect against some Gram-positive bacteria strains (*S. aureus*, *Listeria inoccua*) and yeast (*Candida albicans*) and potentially might be used as a natural oral antiseptic rinse.^240^ Recently, the activity of an alginate-enclosed calcium phosphate iron-saturated bLF formulation was evaluated.^241^ The suggested nanocarriers showed promising results in cancer therapy by suppressing the proliferation of Caco-2 cells. The synthesized LF–AuNP nanoconjugate stabilized by polyethylene glycol (PEG) represented an efficient therapeutic agent against glioblastoma (GBM).^242^ Additionally, the treatment was assisted with photothermal therapy (PTT) laser irradiation. The proliferation of cancer cells significantly reduced resulting in targeting of LF–PEG–AuNP through LF receptors of GBM tissue.

It is worth remembering that the selection of the appropriate delivery carrier is directly influenced by numerous factors, such as the target site of action and the route of drug administration.

LF has been widely known by strong bactericidal and bacteriostatic properties against numerous pathogenic microorganisms. It is worthwhile to underline that the mechanism of LF antibacterial activity is closely related to its iron saturation level. For instance, the populations of Gram-negative strains of *E. coli* and *Klebsiella pneumoniae* were reduced as the LF form was less saturated with iron.^20^ Additionally, Dionysius et al. reported that iron-free LF and native LF efficiently inhibited the growth of *E. coli* strains, whereas the bacterial treatment with holo-LF showed no satisfactory result.^243^ A similar tendency was observed for *Pseudomonas syringae*, whose growth inhibition was dose dependent and required 0.9, 7.5, and 15 mg/mL apo-LF, native LF, and holo-LF, respectively.^244^ In contrast, none of the supplemented bLF forms (native LF, apo-LF, holo-LF) exhibited an evident antibacterial effect against Gram-negative *S. aureus* strains.^20^ Remarkably, Lu et al. pointed out that both the apo and holo forms were able to inhibit the viability of *Streptococcus agalactiae* species, indicating the possibility of LF to act through iron-dependent and iron-independent mechanisms.^245^

Iron is commonly known an essential element required in the formation of bacteria membranes and regulation of their growth. Generally, two mechanisms of iron acquisition by microorganisms are recognized. The first one relies on siderophore secretion by microorganisms which are highly susceptible to iron chelating and following the transport of such a complex across the outer membrane into the cell. For example, *E. coli* bacteria tend to produce aerobactin-type siderophores which are characterized by high binding affinity to iron.^243^ The other proposed mechanism concerns the interactions between microorganism receptors and iron-saturated proteins from the transferrin family, e.g. TF and LF.^246,247^ The iron-dependent (bacteriostatic) antimicrobial mechanism is mainly based on the high iron-chelation potential of LF and the limitation of the availability of that element; thus it provides inhibition of bacteria growth.^248^ The protein affinity to ferric ions tends to decrease as the saturation level of LF increases; thus the described mechanism is considered inefficient in the case of holo-LF. Besides, the bacteriostatic activity of LF might be interrupted after high-temperature treatment (over 75 °C), leading to protein destruction and the loss of the ability to bind iron.^16^

Another bactericidal mechanism is caused by direct interactions between the LF molecule and the microorganism, which in consequence leads to bacteria death.^249^ Ellison et al. revealed a high binding affinity of a positively charged LF molecule to the outer membrane components of bacteria, in particular, negatively charged lipopolysaccharides (LPS) found in Gram-negative bacteria.^250^ As usual, such interactions have a destabilization character, providing release of the LPS and following destruction of the cell membrane. Besides, Duarte et al. pointed that such binding might be disrupted in the presence of high concentrations of divalent cations (Fe^2+^, Cu^2+^, Zn^2+^, Mn^2+^) which will compete with LF for potential binding sites on the surface of bacteria cells.^251^ Remarkably, LF nanoparticles produced by complexation with gellan gum showed a higher antimicrobial activity against *S. aureus* strains in comparison to native LF, probably due to stronger electrostatic interactions with LPS.^251^

In recent time, the greatest attention has been paid to peptides derived from LF enzymatic hydrolysis, e.g. lactoferricin B (LFcin B) and lactoferrampin (LFampin). The derived peptides might be characterized by the same or even higher biological potential in comparison to native protein. Interestingly, such high antibacterial activities of LF peptides are not influenced by iron sequestering but rather by direct interactions with bacteria membranes (bactericidal) leading to the destabilization of their structures. LFcin B belongs to one of the most known peptides of LF which was identified between 17 and 41 residues in the N-terminal region.^66^ Inhibition of bacteria growth in the presence of LFcin B was considered even more effective than with untreated LF. Moreover, Dionysius et al. reported that peptide 1 (pep1) located in the same region as LFcin B with one more additional amino acid (17–42) showed 3 times higher antimicrobial properties toward *E. coli* strains compared to native protein.^252^ Another active fragment of lactoferrin, known as lactoferrampin (LFampin), might be obtained as a product of LF hydrolysis.^253^ LFampin belongs to the cationic amphipathic peptides (268–284) located in the N_1_-domain of bLF. The peptide structure is divided into two major regions: the hydrophobic N-amphipathic helix and the positively charged C-terminal cluster. The hydrophilic fragment is a key element of the hydrolysate since it initiates an antimicrobial mechanism against Gram-positive and Gram-negative pathogens. The neutralization of LFampin’s net charge tends to reduce its permeability through the bacteria membrane, which corresponds with the immediate loss of biological activity.^254^ Van Der Kraan et al. showed a great bactericidal effect of LFampin against series of bacteria strains, e.g. *E. coli*, *P. aeruginosa*, and *Bacillus subtilis*.^255^ Importantly, that analyzed hydrolysate was characterized by higher antibacterial activity in comparison with that of native protein. The authors also related a strong bactericidal effect of these peptides to the electrostatic character of binding with bacteria membrane. The described investigations prove a promising perspective of LF as an efficient antibacterial agent for biomedical applications.

Previous works indicated strong antiviral properties of LF against several dangerous microorganisms. The fundamental mechanism is based on the specific interactions of LF with host cell receptors as well as virus particles, preventing entry of the last one into the cytoplasm environment.^232^ Superti et al. reported the highest effect of bLF on rotavirus inhibition during the initial step of infection (adhesion).^151^ On the other hand, Ikeda et al. did not observe any activity of bLF against the hepatitis C virus (HCV) after the absorption of the virus into the cell.^126^ The type of chelated metal as well as saturation level may contribute to the protein antiviral potential. Indeed, Puddu et al. revealed the greater efficacy in the case of treatment with Me–LF complexes, which might be related to their stronger interactions with LF receptors located on host cells.^233^ The presence of Fe^3+^ ions led to much higher LF antiviral activity in comparison to Mn^2+^ and Zn^2+^ ions. Superti et al. also had noticed a pH-dependent character of binding between bLF and the influenza virus.^256^ The authors revealed that bLF treatment against a viral infection was the most effective at acidic conditions (pH 4–5). In the last years, searching for potential drug candidates against severe acute respiratory syndrome coronavirus-2 (SARS-CoV-2) was extremely topical. LF, widely known as a strong immunomodulator, was not beyond the testing. Conducted studies have showed the ambiguous effect of the protein in the treatment and prevention of COVID-19. The proposed antiviral mechanism of LF against SARS-CoV-2 was comparable to those against other viral infections. It was assumed that LF mediates in the binding with negatively charged molecules of heparan sulfate proteoglycans (HSPGs) found on the cell surfaces of host cells and thereby prevents the viral entry.^257^ Despite the inhibition of the viral replication, LF significantly reduced the level of pro-inflammatory cytokines, such as IL-1β and IL-6.^258^ On the contrary, *in vivo* results were not satisfactory enough. According to a recent clinical trial, bLF supplementation (600 mg/day) did not ensure the total protection of the care personnel of a hospital against SARS-CoV-2 infection.^259^ SARS-CoV-2 symptoms were confirmed in 11 out of 104 participants (10.6%). Nor was any evident effect observed during a 30 day treatment of SARS-CoV-2 infected patients with a daily dose of 800 mg of bLF.^260^ Thus, the practical application of bLF against COVID-19 requires more clinical testing, taking into account the individual characteristics of each patient.

LF’s antioxidant potential is questionable according to controversial results obtained from various research. It has been reported that the free-radical scavenging properties of LF are highly affected by the Fe^3+^ saturation level. Belizi et al. pointed out that holo-LF exhibited 15.4% lower antioxidant activity in comparison to apo-LF.^261^ It is widely known that ferrous ions (Fe^2+^) in aqueous medium are capable of inducing the formation of ROS, as a result of the Fenton reaction (4).
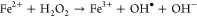
4

Excessive production of ROS leads to a prooxidative/antioxidative imbalance in the organism followed by oxidative stress. Miller et al. have explained the importance of iron coordination by strong ligands, e.g. EDTA and DTPA.^262^ In our case, apo-LF acts as a chelating agent forming a stable Fe–LF complex. Recently, it has been revealed that interactions of apo-LF with red blood cell receptors led to the reduced production of ROS inside the cell in comparison to holo-LF.^263^ The authors assumed the great influence of the free binding sites of apo-LF in prevention of the Fenton reaction, while the completely saturated bLF was not able to chelate free ferric ions. In addition, antioxidative properties of hLF are also determined by overexpression of antioxidative enzymes (SOD1, GP_X_, PRDX), which are known as the first line of host defense against oxidative stress in the initial stages.^219^ bLF supplementation allows prevention of lipid peroxidation developed by chronic hepatitis C.^264^ The level of 8-isoprostane, which is considered a biomarker of oxidative stress, was reduced after bLF treatment, and its concentration in plasma was not correlated with iron metabolism. Condurache et al. pointed out that LF enzymatic digestion led to the release of bioactive peptides with greater antioxidant activities in comparison to native protein.^265^ The antioxidant potential of the obtained hydrolysates was positively correlated with the time of proteolysis. On the contrary, ROS generation inside macrophages promotes phagocytosis activation and inhibits the growth of parasites.^263^ In particular, holo-LF isolated from human neutrophils enhanced the production of highly reactive hydroxyl radicals. A similar mechanism was observed in the presence of iron-saturated transferrin. On the other hand, the amount of hydroxyl radical derived from another family of iron-saturated proteins, e.g. ferritin and desferrioxamine, was insignificant.^266^ Such an effect is probably induced by the high sequence similarity between TF and LF. Indeed, unsaturated forms of TF and LF are capable of specifically interacting with ferrous ions (Fe^2+^) by generation of some ROS, including hydroxyl radicals (OH^•^) and hydrogen peroxide (H_2_O_2_).^267^ Importantly, bLF exhibited a damaging effect in leukemia cell lines by the enhanced production of ROS and activation of caspase mediators in the mitochondrial pathway of apoptosis.^268^

Metal-based complexes of LF have great potential as nutritional additives, especially in dairy products. A number of studies have confirmed a great antibacterial effect of LF against some pathogenic microorganisms (*E. coli*, *K. pneumoniae, P. syringae*).^20^ Indeed, it has been reported that the addition of LF stimulates the growth of beneficial bacterial strains—probiotics—such as *Lactobacillus* and *Bifidobacterium* (*L. plantarum*, *L. paracasei*, *L. rhamnosus*, *B. longum*, *B. bifidum*, *B. infantis*, *B. breve*).^20,269^ Fermented dairy products (kefir, yogurt) represent a rich source of probiotics whose regular consumption improves gut health. Importantly, the prebiotic activity of bLF might be highly affected by the source of isolation. bLF isolated from bovine milk showed a high growth promotion activity which was positively correlated with the protein concentration. On the contrary, the prebiotic properties of colostral bLF were irrelevant.^270^ Glycosylation heterogeneity is one of the key factors which might entail the difference in prebiotic activity of bLF from milk and colostrum. Remarkably, *N*-glycans enzymatically released from whey proteins provided more significant growth of *B. infantis* in comparison to native glycosylated biomolecules.^271^ Thus, free glycans represent a more available carbon source for bacteria growth and provide for their much higher probiotic activity than carbohydrates linked to macromolecules. Interestingly, iron accessibility does not cause any significant difference in the growth promotion of *Bifidobacterium* strains.^270^ The combination of probiotics and lactoferrin represents a novel strategy of food supplements which promotes a healthier gut environment and decreases the risk of the gastrointestinal infections. For example, Chen el al. confirmed a synergistic effect of bLF and its hydrolysates together with probiotic bacteria strains (*L. fermentum*, *B. longum*, *B. lactis*) against foodborne pathogens (*E. coli*, *Salmonella typhi*, *Salmonella typhimurium*).^272^ The authors underlined that the appropriate combination of an LF form and a probiotic is required to avoid the growth inhibition of the beneficial bacteria. Similar studies proved that the supplementation of apo-LF with *L. fermentum* was the most optimal against meticillin-resistant *Staphylococcus aureus* (MSRA) infection.^273^ Besides, the recent studies reported the ability of an LF–probiotic–oligosaccharide mixture to modulate the expression of cytokines; thus such preparations might be efficient for the treatment of the abundant neonates’ intestinal disease, such as necrotizing enterocolitis (NEC).^274^ Similarly, the clinical research confirmed that bLF supplementation in combination with probiotics showed a beneficial effect in the prevention of invasive fungal infections (IFI) in newborns.^275^ Thus, based on the promising results, the combination of LF and a probiotic might represent a highly perspective approach for the application in infant formula.

Another significantly important feature of bLF is enzymatic activity. It was noticed that LF can act in the same way as peroxidase, amylase, phosphatase, protease, and ATPase; however, its activity was relatively lower compared to those of standard enzymes and was highly influenced by the breed of cow.^276^ The authors related the difference in enzymatic activity to the glycosylation level of bLF. Glycan chains could be potential active centers which participate in catalysis. Diversity of glycosylation in the cow’s milk may specifically modify the enzymatic activity of bLF.

bLF effectively acts as an anti-inflammatory agent, which is capable of interacting with immune cells and signaling molecules and controlling the expression of pro-inflammatory and anti-inflammatory genes. NF-κB is one of the major inflammation biomarkers, which stimulates the secretion of pro-inflammatory cytokines.^277^ LPS are the driving molecules which participate in the activation of the NF-κB pathway, immediately after bacterial infection. Accordingly to the bactericidal mechanism, LF tends to interact strongly with LPS of Gram-negative bacteria, in turn preventing the activation of the NF-κB pathway.^79^ The anti-inflammatory potential of bLF is mainly contributed by its capacity to bind with macrophage receptors providing changes in plasma cytokine expression. The level of pro-inflammatory cytokines (interleukin-6, IL-6; interleukin-1β, IL-1β; tumor necrosis factor α, TNF-α) was surpassed after bLF treatment, while the expression of anti-inflammatory cytokine (IL-10) increased.^278^ The nonsaturated form of LF revealed a higher inhibitory effect against pro-inflammatory cytokines.^243^ LF interaction with epithelial cells modulates the expression of pro-inflammatory cytokines.^279^ The concentration of IL-8 was significantly reduced resulting in treatment of infected cells by *E. coli* with native-bLF and holo-bLF.

bLF plays a significant role in stimulation of the human immune system, particularly by interactions with immune system cells. LPS are known as characteristic outer membrane components of Gram-negative bacteria which are also capable of binding with LF and activating the innate immune response.^280^ The high level of LPS leads to overproduction of ROS in neutrophils and cell damage.^281^ LF is able to compete with neutrophils for LPS binding. The complexation of LPS by LF allows inhibition of superoxide radical production in neutrophils and prevents oxidative stress.^282^ LF is capable of modulating the activity of lymphocytes and regulating the adaptive immune response by the level of expressed antibodies. Moreover, LF participates in the activation of the TLR receptors signaling pathway.^263^ TLR receptors are localized in macrophages, fibroblasts, dendritic cells, and epithelial cells.^283^ bLF glycosylation has an impact on the nuclear factor κB (NF-κB) signaling pathway, which allows modulation of the therapeutic properties of protein. Heterogeneity of *N*-glycan chains in bLF induces the diverse effect on NF-κB expression. Cell treatment with the desialated LF form provided a lower release of NF-κB in TLR receptors compared to sialated bLF. In contrast, demannosylation of bLF corresponded to activation of NF-κB production.^284^ It can be assumed that sialic acid acts as a mediator in signal recognition via TLR receptors providing release of NF-κB, while the interactions between mannose residues and TLR are not favorable. Moreover, bLF supplementation promotes the expression of total (CD3^+^), helper (CD4^+^), and cytotoxic (CD8^+^) T-cell activations which regulate cytokine secretion.^285^

In recent years, LF has attracted wide attention in the scientific community as a potential anticancer agent. Many previous studies confirmed that LF is capable of inhibiting the growth and metastasis of tumor. In many cases, uncontrolled plasminogen activation becomes the reason for tumor cell invasion.^286^ The positively charged N-terminal fragment of LF is capable of inhibiting plasminogen binding to the host cell surface. This interaction precludes plasminogen transformation into plasmin by the urokinase-type plasminogen activator.^287^ bLF treatment led to prominently reduced proliferation of canine mammary tumor cells (CIPp, CHMp).^288^ The experimental finding confirmed that the antitumor effect of LF was highly affected by the concentration of added protein. Interestingly, LF oligomers also might be sufficient in cancer treatment. It has been reported that high molecular weight LF (HMW-LF) induced a cytotoxic effect against human breast carcinoma and human colorectal adenocarcinoma cell lines.^15^ Ebrahim et al. reported that HWM-LF at the concentration of 3200 μg/mL showed the highest cytotoxic effect against human breast carcinoma (MDA-MB-231) and human colorectal adenocarcinoma (SW480) in comparison to native LF, apo-LF, and holo-LF with the same concentration.^15^ The greater antitumor potential of HMW-LF might be caused by enhanced activity of caspase-3 enzyme mediated in the mitochondrial pathway of tumor cell death (apoptosis). On the other hand, the fairly difficult question arises of how to maintain LF biological activity during gastric digestion. In recent times, the milk proteins have been widely studied in cancer prophylaxis. Sakai et al. showed that enzymatically obtained bLF peptides also showed cytotoxic activity against human oral squamous carcinoma cell line (SAS).^289^ The efficiency of potential anticancer agents was estimated by monitoring the release of the LDH marker in tumor cells. Generally, LDH overexpression indicates that cancer invaded living cells.^290^ In addition, a synergetic cytotoxic effect of a whey biomolecule combination of LPO and LF in breast cancer cell lines (MCF-7, MDA) has been proven.^291^ The relationship between the metal saturation level and LF anticancer potential has not been clearly determined so far. For, example, Zhang et al. suggested that Fe–LF addition causes ROS overproduction, facilitating the apoptosis (ferroptosis) of tumor cells.^173^

The biological activity of LF could be influenced by the presence of LF receptors (LFR) in some host cells. LF is capable of interacting with various types of cells. Many studies confirmed that LFR are located on the surface of monocytes, macrophages, lymphocytes, erythrocytes, hepatocytes, enterocytes, dendritic cells, and epithelial cells. It was observed that the activity of LFR in respiratory epithelial cells was enhanced with the exposure of metals (Fe^3+^, VO^2+^), which suggests that LFR could be involved in a detoxication process and reduction of oxidative stress.^292^ Some pathogens, including different species of the Neisseriaceae and Pasteurellaceae families, contain iron–glycoprotein receptors on their surfaces which directly interact with LF. In turn, iron is taken up by pathogens, stimulating their growth.^293,294^ LbpA and LbpB genes encode the LF receptor in bacterial membrane.^295^ It has been reported that LF–receptor interactions occur preferentially by binding between the LF N-lobe and the LbpB C-lobe, whether LbpA participates in the metal uptake process.^247^ On the other hand, the binding affinity of these receptors becomes limited in the medium of iron excess. Interactions of LF with monocyte cells provided a slight decrease of the protein isoelectric point, followed by reducing the affinity for immune receptors.^296^ Based on this, it was suggested that electrostatic type interactions through positively charged amino acids are favored between LF and monocytes. Interestingly other parameters of LF, like molecular weight and iron-binding affinity, were maintained. Thus, LF interaction with monocytes concerned exclusively the availability of basic amino acids on the protein surface not leading to other modifications in the LF structure.

## 10 Future Perspectives

Lately, LF attracts more attention due its multifunctionality. This review described the major features of the LF molecular structure, such as the tendency to iron chelation and glycosylation variations. The development of an LF isolation method is currently focused on finding the universal highly efficient approach that will be compatible for different types of raw materials. The combination of chromatographic and membrane techniques has the potential to become the most optimal approach in LF production due to the high purity level of the final product and the maximum recovery. Importantly, the separation conditions (pH, temperature, ionic strength, pressure, drying method) have a considerable impact on the protein conformational stability; thus they should be strictly controlled to prevent the loss of LF biological activity. The appropriate analytical tools, including spectroscopic, spectrometric, chromatographic, and electromigration techniques, provide a comprehensive analysis of the LF physicochemical properties (molecular weight, isoelectric point, iron saturation degree, glycosylation patterns). Since LF is naturally found as a mixture of apo-LF and holo-LF, a more advanced instrumental approach is required for the distinction and quantification of each individual form in native protein. Besides, the detailed insights into the MALDI-TOF/MS technique is essential for understanding the relationship between the LF glycosylation heterogeneity and the protein biological activity.

The remarkable health benefits of LF indicate its wide range of potential applications, including in dietary supplements, infant formula, the food industry, and medicine. LF and its related peptides (LFcin, LFampin) are commonly known for their strong antibacterial properties, and they might be used as natural food preservatives.^297^ Despite a high nutritional value, the products fortified in LF will be characterized by an extended shelf life. LF’s binding capacity to numerous essential biomolecules, such as microelements, fatty acids, and polyphenols, indicates its promising delivery properties. Experimental findings showed that the supplementation of holo-LF facilitates the metal absorption by intestinal mucosa and might be efficient in the treatment of iron deficiency diseases.^298^ The greatest demand for such LF preparations is predicted for groups of people with the poorest iron bioavailability, such as neonates, pregnant women, vegetarians, and older adults. The ability of LF to boost the immune system and stimulate the growth of beneficial bacteria (probiotics), e.g. *Lactobacillus* and *Bifidobacterium*, could be especially topical for infant formula.^20^ Remarkably, LF in combination with probiotics might act synergistically, protecting the gut microenvironment against pathogens more effectively. Besides, one of the key fields of LF consumption is medical treatment. The high efficiency of LF against a number of bacterial and viral infections has been proven. Additionally, recent *in vitro* studies have introduced the antiviral mechanism of LF against SARS-CoV-2. Remarkably, LF supplementation also has demonstrated promising results in cancer therapy. Nevertheless, the major problem of the delivery of LF to the target site of action is related to the sensitivity of the protein structure, especially in gastrointestinal conditions. In order to prevent LF degradation, various delivery strategies have been developed. The encapsulation of LF by using natural-based materials, e.g. polysaccharides (chitosan, alginate, pectin, gum Arabic, heparin), lipids (phospholipids, cholesterol, soy lecithin), and proteins (caseins, whey proteins, gelatin), has become one of the fast-growing trends used for improving protein bioavailability.^299,300^ Although LF is considered entirely safe for humans, more detailed clinical trials are required before its inclusion in medicinal products.

## Abbreviations Used

ACE: affinity capillary electrophoresis
AgNPs: silver nanoparticles
AMF: anhydrous milk fat
ATR: attenuated total reflection
AuNPs: gold nanoparticles
bLF: bovine lactoferrin
BOD: biological oxygen demand
bTF: bovine transferrin
CA: carrier ampholyte
CD: circular dichroism
CE: capillary electrophoresis
CGA: chlorogenic acid
cIEF: capillary isoelectric focusing
CM: carboxymethyl
CN: casein
COD: chemical oxygen demand
COVID-19: coronavirus disease 2019
DEAE: diethylaminoethyl
DHB: 2,5-dihydroxybenzoic acid
DLS: dynamic light scattering
DTPA: diethylenetriaminepentaacetic acid
DSC: differential scanning calorimetry
EDA: ethylenediamine
EDTA: ethylenediaminetetraacetic acid
ELISA: enzyme-linked immunosorbent assay
EOF: electroosmotic flow
ESI: electrospray ionization
Fe-NTA: ferric nitrilotriacetate
FTIR: Fourier-transform infrared
Fuc: fucose
Gal: galactose
GalNAc: *N*-acetylgalactosamine
GlcNAc: *N*-acetylglucosamine
GBM: glioblastoma
GPC: gel permeation chromatography
GPx: glutathione peroxidase
GR: glutathione reductase
GST: glutathione-*S*-transferase
HA: hyaluronic acid
HCV: hepatitis C virus
hLF: human lactoferrin
HMW: high molecular weight
HPLC: high-performance liquid chromatography
HSV: herpes simplex virus
hTF: human transferrin
HTST: high temperature–short time
ICP-MS: inductively coupled plasma mass spectrometry
IEF: isoelectric focusing
IFI: invasive fungal infections
Ig: immunoglobulins
IL-1β: interleukin 1β
ITC: isothermal titration calorimetry
LAB: lactic acid bacteria
LC: liquid chromatography
LDH: lactate dehydrogenase
LF: lactoferrin
LFampin: lactoferrampin
LFcin: lactoferricin
LFR: lactoferrin receptor
LMCT: ligand–metal charge transfer
LPO: lactoperoxidase
LPS: lipopolysaccharides
MALDI-TOF/MS: matrix-assisted laser desorption/ionization time of flight mass spectrometry
Man: mannose
MD: molecular docking
MES: 2-(*N*-morpholino)ethanesulfonic acid
MF: microfiltration
MRM: multiple reaction monitoring
NEC: necrotizing enterocolitis
Nd:YAG: neodymium-doped yttrium aluminum garnet
NeuAc: *N*-acetylneuraminic acid
NF: nanofiltration
NF-κB: nuclear factor κB
pI: isoelectric point
PMF: peptide mass fingerprint
OA: oleic acid
PDB: Protein Data Bank
PEG: polyethylene glycol
PRDX: peroxiredoxin
PTT: photothermal therapy
PVA: poly(vinyl alcohol)
RO: reverse osmosis
ROS: reactive oxygen species
rTF: rabbit transferrin
SA: serum albumin
SARS-CoV-2: severe acute respiratory syndrome coronavirus 2
SDS-PAGE: sodium dodecyl sulfate polyacrylamide gel electrophoresis
SEC: size-exclusion chromatography
SERS: surface enhanced Raman spectroscopy
SOD1: superoxide dismutase 1
SP: sulfopropyl
TEM: transmission electron microscopy
TF: transferrin
TFA: trifluoroacetic acid
TFF: tangential flow filtration
TNF-α: tumor necrosis factor-α
UF: ultrafiltration
UHT: ultrahigh temperature
UV–vis: ultraviolet–visible
WPs: whey proteins
XRD: X-ray diffraction
2D-PAGE: two-dimensional polyacrylamide gel electrophoresis
